# Improved supervised prediction of aging-related genes via weighted dynamic network analysis

**DOI:** 10.1186/s12859-021-04439-3

**Published:** 2021-10-25

**Authors:** Qi Li, Khalique Newaz, Tijana Milenković

**Affiliations:** Department of Computer Science and Engineering, Center for Network and Data Science (CNDS), and Eck Institute for Global Health, University of Notre Dame, Notre Dame, IN 46556 USA

**Keywords:** Biological networks, Dynamic network analysis, Aging, Node classification

## Abstract

**Background:**

This study focuses on the task of supervised prediction of aging-related genes from -omics data. Unlike gene expression methods for this task that capture aging-specific information but *ignore interactions* between genes (i.e., their protein products), or protein–protein interaction (PPI) network methods for this task that account for PPIs but the PPIs are *context-unspecific*, we recently integrated the two data types into an aging-specific PPI subnetwork, which yielded more accurate aging-related gene predictions. However, a *dynamic* aging-specific subnetwork did not improve prediction performance compared to a *static* aging-specific subnetwork, despite the aging process being dynamic. This could be because the dynamic subnetwork was inferred using a naive Induced subgraph approach. Instead, we recently inferred a dynamic aging-specific subnetwork using a methodologically more advanced notion of network propagation (NP), which improved upon Induced dynamic aging-specific subnetwork in a different task, that of *unsupervised* analyses of the aging process.

**Results:**

Here, we evaluate whether our existing NP-based dynamic subnetwork will improve upon the dynamic as well as static subnetwork constructed by the Induced approach in the considered task of *supervised* prediction of aging-related genes. The existing NP-based subnetwork is unweighted, i.e., it gives equal importance to each of the aging-specific PPIs. Because accounting for aging-specific edge weights might be important, we additionally propose a *weighted* NP-based dynamic aging-specific subnetwork. We demonstrate that a predictive machine learning model trained and tested on the weighted subnetwork yields higher accuracy when predicting aging-related genes than predictive models run on the existing unweighted dynamic or static subnetworks, regardless of whether the existing subnetworks were inferred using NP or the Induced approach.

**Conclusions:**

Our proposed weighted dynamic aging-specific subnetwork and its corresponding predictive model could guide with higher confidence than the existing data and models the discovery of novel aging-related gene candidates for future wet lab validation.

**Supplementary Information:**

The online version contains supplementary material available at 10.1186/s12859-021-04439-3.

## Introduction

### Motivation and related work

Incidence of many complex diseases, such as diabetes, cancer, osteoarthritis, cardiovascular, and Alzheimer’s disease, increases with age [1]. Even recent and widespread COVID-19 is highly related to aging. Namely, according to the United States Centers for Disease Control and Prevention^1^, as of March 24, 2021, ${\sim }\,81\%$ of all deaths related to COVID-19 occurred in the age range of 65 years and above.

Understanding the molecular mechanisms behind the aging process, including comprehensive and accurate identification of human genes implicated in aging, is important for studying and treating such aging-related diseases [2, 3]. However, analyzing human aging via wet lab experiments is difficult due to long human life span and ethical constraints [4, 5]. Analyzing human aging computationally can fill this gap. This includes identification (i.e., prediction) of aging-related genes via supervised learning from human -omics data [3, 6, 7], which is the task that we focus on in this paper. By supervised prediction of aging-related genes, we mean that a part of current aging-related knowledge is used when predicting which genes are linked to aging, and the remaining part of that knowledge is used when evaluating the predictions. On the other hand, an orthogonal and thus complementary task, which is outside of the scope of this paper, is that of unsupervised prediction of aging-related genes. By this, we mean that no current aging-related knowledge is used when making predictions, and instead, all of the aging-related knowledge is used only to evaluate the predictions.

Approaches for our task at hand—supervised prediction of aging-related genes—can be categorized as follows: (1) those that use gene expression data alone, (2) those that use protein–protein interaction (PPI) network data alone, and (3) those that combine gene expression data with PPI network data.

Approaches from the first category predict a gene as aging-related if its expression level varies with age [8–12]. While such approaches do capture aging-specific information, they ignore interactions between genes, i.e., their protein products. This is their drawback, because proteins carry out cellular functioning, including the aging process, by interacting with each other [13].

Approaches from the second category predict a gene as aging-related if its position (i.e., node representation/embedding/feature) in the PPI network is “similar enough” to the network positions of known aging-related genes [6, 7, 14, 15]. While these approaches do consider PPIs that carry out cellular functioning, their drawback is that the PPIs are context-unspecific, i.e., the PPIs span different conditions, such as cell types, tissues, diseases, environments, or patients. In the context of our study, this means that the PPIs are not aging-specific, i.e., they span different ages.

Approaches from the third category address the above drawbacks by considering both aging-specific gene expression data and context-unspecific PPI network data. Earlier approaches of this type extracted genes’ features from each of gene expression data and PPI network data individually and then concatenated the features [6, 7]. As such, they integrated the features rather than the data. Consequently, they still considered the context-unspecific PPI network. More recent efforts, including by our group, first integrated the two data types to infer an aging-specific subnetwork of the entire context-unspecific PPI network, and then analyzed the resulting subnetwork [4, 16]. Specifically, these studies used a traditional *Induced approach*: given gene expression data for different ages and an entire context-unspecific PPI network (which happens to be *static*), the studies formed a subnetwork snapshot for each age, where each snapshot consisted of all genes that were significantly expressed (i.e., active) at the given age and all PPIs from the entire network that exist between the active genes. That is, they formed a given age-specific snapshot by taking the Induced subgraph on the active genes at that age. Then, they combined snapshots for all ages into a *dynamic* aging-specific PPI subnetwork. Such a subnetwork, being dynamic, is meant to capture how network positions of genes change with age.

These studies used their inferred dynamic subnetworks in *unsupervised* aging-related tasks [4, 16]. So, while their inferred subnetworks are relevant for this paper, their unsupervised analyses of the subnetworks fall outside the scope of this paper.

On the other hand, *supervised* analyses from our other, even more recent work [17, 18] *are* directly relevant for this paper. In that work, we performed supervised prediction of aging-related genes from three networks: (i) the dynamic aging-specific subnetwork inferred by Faisal and Milenković [4], (ii) a static (still aging-specific) counterpart of this dynamic subnetwork, and (iii) the entire (also static) context-unspecific PPI network from which the above dynamic aging-specific subnetwork was inferred. Note that the static Induced subnetwork from point ii) was constructed by aggregating all nodes and edges over all snapshots of the dynamic Induced subnetwork. Then, first, we examined whether using the dynamic or static aging-specific subnetwork improved the supervised prediction accuracy compared to using the entire (static) context-unspecific PPI network. Indeed, we found this to hold overall (i.e., it held for many although not all of the considered evaluation measures). Second, because the aging process is dynamic, we examined whether using the dynamic aging-specific subnetwork was superior to using the static aging-specific subnetwork. Surprisingly, we found this *not* to hold overall (i.e., it did not hold for most of the considered evaluation measures).

This unexpected finding could be because the dynamic subnetwork was inferred using the Induced approach, which is quite naive as it considers *all* PPIs from the context-unspecific network that exist between *only* the active genes at a given age. However, first, not all PPIs between the active genes might be equally “important”, and the Induced approach has no way of identifying the most important of all such PPIs [19]. Second, the Induced approach fails to consider any inactive genes that might critically connect the active genes in the network [19].

Recently, an alternative, methodologically more advanced notion of *network propagation (NP)* was used for inference of a *static* context-specific subnetwork in the context of *cancer* [20]. NP maps expression levels (i.e., activities) onto the genes in the entire context-unspecific PPI network. Then, NP propagates the activities via random walk or diffusion, to assign condition-specific weights to the nodes (genes, i.e., their protein products) or edges (PPIs) in the entire PPI network. Finally, NP assumes that it is the highest-weighted network regions that are the most relevant for the condition of interest, i.e., such regions form the context-specific subnetwork. Hence, as opposed to the Induced approach, first, NP assigns weights to PPIs that can help identify the most “important” PPIs. Second, NP can consider a non-active gene if, for example, the gene is connected to sufficiently many active genes.

Our group extended two prominent NP approaches, i.e., NetWalk [21] and HotNet2 [22], to allow for the inference of *dynamic* context-specific subnetworks in the context of *aging* [19]. Then, we evaluated the NP-based aging-specific subnetworks in an *unsupervised* learning context, finding that NP-based dynamic aging-specific subnetworks outperform the dynamic aging-specific subnetwork created using the Induced approach [19]. In particular, a dynamic aging-specific subnetwork inferred using the NetWalk method outperformed every other considered dynamic aging-specific subnetwork [19]. This is why in this paper, out of all existing NP-based aging-specific subnetworks, we focus on the NetWalk-based one. For methodological details of NetWalk and HotNet2, please refer to their original publications [21, 22] or to our work on unsupervised study of aging [19]. Note that while the inferred NP-based subnetworks are relevant for this paper, the unsupervised analyses of the networks by Newaz and Milenković [19] fall outside of its scope.

With the above information in mind, in this paper, a key question that we aim to answer is whether using an NP-based dynamic aging-specific subnetwork (either the existing one by NetWalk or a novel one that we propose as a contribution of our study; see below) will outperform every other available network (i.e., the dynamic and static aging-specific subnetworks resulting from the Induced approach, static counterparts of the dynamic NP-based aging-specific subnetworks, and the entire static context-unspecific PPI network). This question has several subquestions, as discussed next.

### Our study and contributions

Our first subquestion is whether using an NP-based dynamic aging-specific subnetwork improves the *supervised* prediction of aging-related genes compared to using any of the existing (dynamic or static) Induced aging-specific subnetworks. To answer this, we consider two NP-based dynamic subnetworks and two Induced subnetworks, as follows.

One of the considered NP-based dynamic aging-specific subnetworks, which we refer to as *NetWalk-Dynamic*, was inferred in our existing study [19] (see *“Methods”*). This subnetwork was constructed by using NP (specifically, NetWalk) to propagate expression activities onto the genes in the entire context-unspecific PPI network in order to assign aging-specific weights to the PPIs. However, NetWalk does not have a procedure of extracting a context-specific (i.e., aging-specific) subnetwork from such a weighted PPI network [19]. Therefore, in our previous study [19], we first applied a weight threshold and then treated all PPIs with weights above the threshold as aging-specific. Then, we extracted the aging-specific PPIs (and proteins involved in these PPIs) as the resulting aging-specific subnetwork. Consequently, this subnetwork is unweighted, as the PPIs were assigned equal importance (see [19] for more detail). This approach, which was the best one could do at the time, suffers from two limitations. First, it is hard to choose a meaningful PPI weight threshold, because there is no clear guideline about which threshold is meaningful. So, one needs to empirically evaluate subnetworks constructed at multiple thresholds, which is computationally inefficient. Second, this approach cannot distinguish between different aging-specific PPIs, because it assumes that each such PPI carries the same amount of aging-specific information as the others. To address these limitations, in this study, we aim to infer a novel, i.e., *weighted* NP-based dynamic aging-specific subnetwork, with hope that accounting for PPI weights, i.e., for aging-specific importance of the PPIs, will perform better in our task of supervised prediction of aging-related genes. We refer to this weighted subnetwork as *wNetWalk-Dynamic* (see *“Methods”*). We aim to evaluate whether at least one of NetWalk-Dynamic and *w*NetWalk-Dynamic is better than the dynamic and static Induced subnetworks from our previous studies that have already been discussed above [17, 18]. We refer to the latter two as Induced-Dynamic and Induced-Static, respectively.

Assuming that NP performs better than the Induced approach, our second subquestion is whether at least one of the two NP-based dynamic aging-specific subnetworks is better than static counterparts of both of the NP-based dynamic subnetworks. We do this because aging is a dynamic process, and so dynamic network analysis of aging should outperform static network analysis of aging. To answer this, for reasons described in “Methods” section, we construct two distinct static counterparts for NetWalk-Dynamic, referred to as NetWalk-Static and NetWalk-Static*, respectively. We also construct a (single) static counterpart for *w*NetWalk-Dynamic, referred to as *w*NetWalk-Static*.

Recall that we hypothesize that *weighted* NP will be better than *unweighted* NP, which is why we infer *w*NetWalk-Dynamic in the first place. So, our third subquestion is to test this hypothesis—whether *w*NetWalk-Dynamic performs better than NetWalk-Dynamic. Note that for as comprehensive evaluation as possible, we also consider the entire context-unspecific network that all aging-specific subnetworks are inferred from, with an expectation that the latter will outperform the entire network. We refer to the entire network as *Entire*. We summarize the eight considered (sub)networks in Table 1.

**Table 1:** Summary of the eight considered (sub)networks for each of two considered entire context-unspecific human PPI networks

Network name	Weighted	Unweighted	Dynamic	Static
Entire		$\checkmark$		$\checkmark$
Induced-Dynamic		$\checkmark$	$\checkmark$	
Induced-Static		$\checkmark$		$\checkmark$
NetWalk-Dynamic		$\checkmark$	$\checkmark$	
NetWalk-Static		$\checkmark$		$\checkmark$
NetWalk-Static*		$\checkmark$		$\checkmark$
*w*NetWalk-Dynamic	$\checkmark$		$\checkmark$	
*w*NetWalk-Static*	$\checkmark$			$\checkmark$

One of the two entire context-unspecific networks is from HPRD [23], which is quite old, but we use it because many relevant aging-specific subnetworks were already inferred from it in the prior work (see text for details). The other entire context-unspecific network is from BioGRID [24], which we use as the most recent human PPI data (“Data” section). So, when we use HPRD, “Entire” represents the HPRD entire context-unspecific network, and the other seven aging-specific subnetworks are all inferred from HPRD. When we use BioGRID, “Entire” represents the BioGRID entire context-unspecific network, and the other seven aging-specific subnetworks are all inferred from BioGRID. We study HPRD (and its associated aging-specific subnetworks) separately from BioGRID (and its associated aging-specific subnetworks), with the goal of evaluating whether results that we observe for HPRD also hold for the most recent BioGRID data

For each (sub)network, we develop multiple supervised predictive models where each model consists of a (1) feature, (2) feature dimensionality reduction choice, and (3) classifier. (4) Regarding features, given that our (sub)networks are of different types (unweighted vs. weighted, and static vs. dynamic), we need to choose features that fit a given network type. For unweighted dynamic and static (sub)networks, we use existing sophisticated features from our previous studies [17, 18]. For weighted dynamic subnetwork, we consider five existing features [25–27]. However, because the existing weighted features are simple extensions of the notion of unweighted network centrality, we believe that we can do better. So, we propose a new way to extract weighted dynamic features of nodes, with hope that they will outperform the existing weighted dynamic features. Thus, our fourth subquestion is whether using our proposed weighted dynamic features will outperform the existing weighted dynamic features on *w*NetWalk-Dynamic. In the process, as a control, we propose static counterparts of our new weighted dynamic features to evaluate whether the latter will be superior, as we would expect. (2) Regarding dimensionality reduction choices, for each feature, we consider its full version and its dimensionality-reduced version. (3) Regarding classifiers, we couple each feature (version) with three prominent classifiers. For details, see Additional file 1: Section S1.

For each predictive model, we evaluate its performance via cross-validation [18]: we train on a subset of known aging- and non-aging-related genes and test on the remaining subset of known aging- and non-aging-related genes. To define known aging- and non-aging-related gene labels for classification, we rely on highly confident ground truth data, i.e., GenAge [28]. We evaluate performance of a predictive model in terms of the area under the precision-recall (AUPR) curve, precision, recall, and F-score. Given all predictive models of a (sub)network, we choose the best predictive model that yields the highest AUPR score. Then, we compare the different (sub)networks, each under its selected best model, via all four prediction accuracy measures. For details, see Additional file 1: Section S1.2.

Because cancer is known to be aging-related [1, 29–31], in addition to cross-validation, we use a highly confident cancer-related data set to validate the performance of each (sub)network. Specifically, given a (sub)network, we measure the number of cancer-related genes among its novel gene predictions, i.e., genes predicted as aging-related that are currently not associated with aging.

**Fig. 1:**
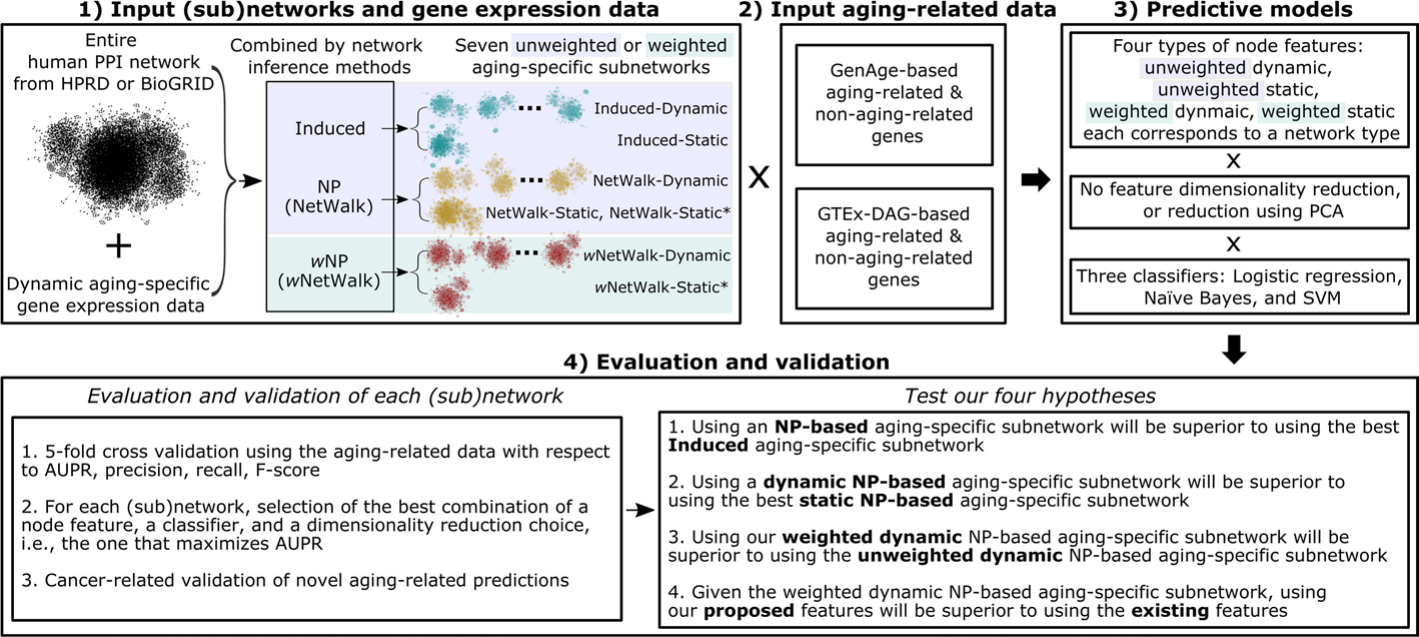
Summary of our study. For details on each step of the study, see the text

In summary, the above four subquestions can be seen as testing the following four hypotheses (for each of HPRD and BioGRID): (1) whether using an *NP-based* aging-specific subnetwork will outperform all *Induced* aging-specific subnetworks, (2) whether using an *NP-based dynamic* aging-specific subnetwork will outperform all *NP-based static* aging-specific subnetworks, (3) whether using the *weighted dynamic* NP-based aging-specific subnetwork will outperform the *unweighted dynamic* NP-based aging-specific subnetworks, and (4) given the weighted dynamic subnetwork, whether using our *proposed* features will outperform *existing* features. We summarize our study in Fig. 1.

We find that overall, all of our four hypotheses hold with respect to both cross-validation and cancer-related validation, for both HPRD and BioGRID. Most importantly, our proposed weighted dynamic aging-specific subnetwork (i.e., *w*NetWalk-Dynamic) along with our proposed weighted dynamic feature performs the best among all considered (sub)networks. This finding justifies the need of using a weighted dynamic aging-specific subnetwork in the task of predicting aging-related genes, which is the key contribution of our study.

Because *w*NetWalk-Dynamic performs the best, we believe that it has potential to guide future experimental validation of its aging-related gene predictions that are currently not known to be aging-related. As the first attempt to this, we identify all six such genes that are predicted only by *w*NetWalk-Dynamic (when using HPRD). We manage to validate all of the six genes by manually searching for a link between them and the aging process in PubMed articles. This finding is another contribution of our study.

## Results

Recall that we consider two entire context-unspecific networks: HPRD and BioGRID. All relevant aging-specific subnetworks from the prior work were inferred from HPRD. For this reason, due to space constraints, in the main paper, we report detailed results primarily for HPRD, but we do discuss BioGRID results briefly as well. We report detailed results for BioGRID in the supplement. Unless otherwise noted, results reported in the main paper are for HPRD.

Moreover, in the main paper, we primarily report detailed results for when using the GenAge-based definition of aging- and non-aging-related gene labels; we do this in the first six subsections. Human genes in GenAge are sequence orthologs of aging-related genes in model species. Because not all human genes have sequence orthologs in model species [32, 33], using GenAge may miss aspects of the aging process that are unique to the human species [34]. So, we consider another source of aging-related knowledge obtained by studying the human species directly (rather than doing so indirectly from model species), namely the down-regulated aging-related genes from genotype-tissue expression (GTEx) project [8], i.e., GTEx-DAG. We report results when using the GTEx-DAG-based label definition in the seventh subsection titled *“Results when using our secondary aging-related gene label definition with respect to GTEx-DAG”*. Note that we consider both GenAge- and GTEx-DAG-based label definitions only for HPRD. For BioGRID, we consider only the GenAge-based definition. This is because analyses on BioGRID are computationally expensive given its large size, and we have had to choose one of GenAge and GTEx-DAG. We have chosen the former because (as we will show) for HPRD-based (sub)networks, prediction accuracy scores are overall much higher when using GenAge than when using GTEx-DAG, which might indicate the former to be of higher quality. Also, results when using GenAge are qualitatively similar for HPRD and BioGRID, and we expect them to be similar when using GTEx-DAG as well.

### The best predictive model for each (sub)network

In this subsection, we comment on how we select the best predictive model for each (HPRD-based) (sub)network. Recall that a predictive model consists of three components, i.e., a node feature, a feature dimensionality reduction choice, and a classifier. Regarding node features, we consider four types of features, each corresponding to one of the four network types (i.e., each feature can only be extracted from its corresponding network type). We summarize the considered features in Table 2. Regarding dimensionality reduction choices, for each feature, we consider its full version as well as its principal component analysis (PCA) reduced versions. Regarding classifiers, for each feature version, we run three classifiers, i.e., logistic regression (LR), naive Bayes (NB), and support vector machine (SVM) with radial basis function (rbf) (SVM-rbf). For methodological details and reasons of why we use these components, see Additional file 1: Section S1. For summary of predictive models we use for each (sub)network, see Additional file 1: Section S1.2 and Additional file 1: Table S1.

**Table 2:** The considered node features and their types, i.e., weighted dynamic, weighted static, unweighted dynamic, and unweighted static

	Weighted	Unweighted
Dynamic	**Diff-nobin-2** DegC-wt, ClusC-wt, CloseC-wt, BetwC-wt, EigenC-wt	DGDV, GoT, GDC, ECC, KC, DegC, CentraMV
Static	**Static-nobin-2**	SGDV, UniNet, 30BPIs

Features in bold are (the best versions of) our proposed features, and the rest are the considered existing features. Note that we list only the best version of our proposed weighted dynamic features for simplicity, as we proposed, tested, and compared 30 such features. Also, note that we only test the weighted static counterpart of the best weighted dynamic feature. For the discussion on our proposed weighted dynamic features, see Section *“The proposed weighted features”*. For the discussion on how we select the best of the existing weighted dynamic features and the best of our proposed weighted dynamic features, see Additional file 1: Section S2 and Additional file 1: Fig. S1

Recall that for each of the eight (sub)networks, we select the best predictive model that yields the highest AUPR score. Briefly, the eight selected models (each corresponding to a (sub)network) encompass five features, two dimensionality reduction choices, and three classifiers (Table 3). While some individual components of a model are somewhat preferred over others, there is no model as a whole that consistently works the best over all types of (sub)networks. So, in summary, there is no clear pattern regarding which component of which model works well on which (sub)network.

**Table 3:** The selected best predictive model for each (sub)network with respect to the GenAge-based aging- and non-aging-related gene labels, and when using HPRD

Entire	Induced-Dynamic	Induced-Static
SGDV + None + NB	DGDV + None + NB	SGDV + None + NB
NetWalk-Dynamic	NetWalk-Static	NetWalk-Static*
DGDV + None + NB	30BPIs + None + SVM-rbf	30BPIs + PCA + LR
*w*NetWalk-Dynamic	*w*NetWalk-Static*	
Diff-nobin-2 + None + LR	Static-nobin-2 + None + LR	

The model is represented as “X+Y+Z”, where X represents the selected feature, Y represents the selected dimensionality reduction choice (i.e., None or PCA), and Z represents the selected classifier. For dimensionality reduction choices, “None” means the selected feature is in its full version, and “PCA” means the selected feature is in its PCA-reduced version. For the best predictive models when using BioGRID instead of HPRD, see Additional file 1: Table S3

### Using NP-based versus induced aging-specific subnetworks

**Fig. 2:**
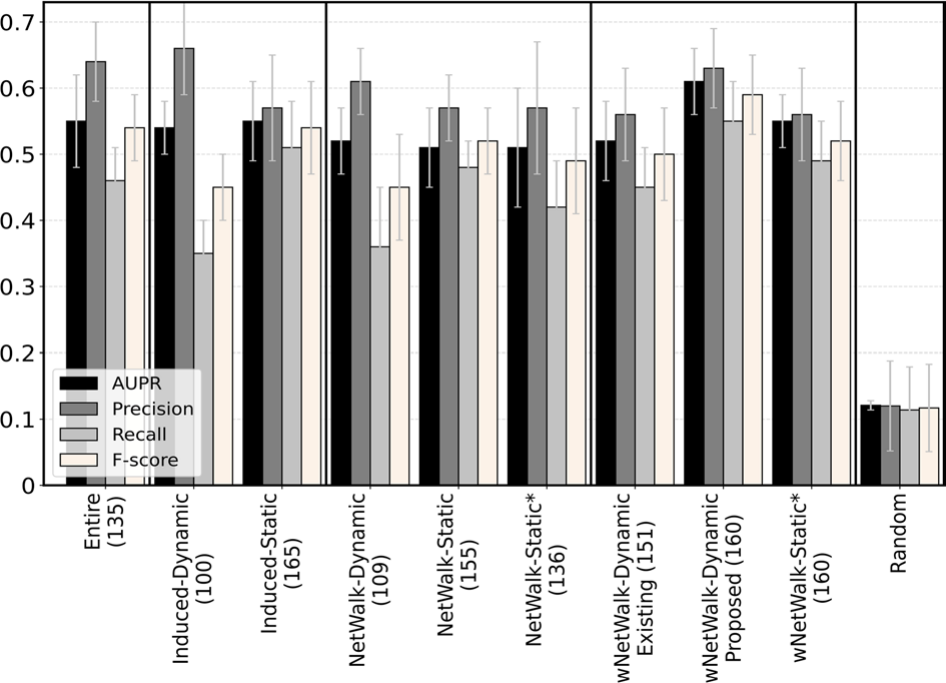
Prediction accuracy in terms of AUPR, precision, recall, and F-score for the eight (sub)networks, each under its best predictive model, when using the primary GenAge-based definition of aging- and non-aging-related gene labels, and when using HPRD. On the *x*-axis, the number in the parenthesis after the name of each (sub)network corresponds to the number of predictions. Note that we have two groups of results for *w*NetWalk-Dynamic, i.e., *w*NetWalk-Dynamic Existing and *w*NetWalk-Dynamic Proposed. The former corresponds to using the best existing weighted dynamic feature on *w*NetWalk-Dynamic, and the latter corresponds to using the best proposed weighted dynamic feature on *w*NetWalk-Dynamic. For results when using BioGRID instead of HPRD, see Additional file 1: Fig. S4

We first comment on our hypothesis (1): whether using an *NP-based* aging-specific subnetwork will outperform all *Induced* aging-specific subnetworks. We begin with testing whether the best of the existing (unweighted) dynamic subnetworks from our previous study [19] (i.e., NetWalk-Dynamic) and its two (unweighted) static counterparts (i.e., NetWalk-Static and NetWalk-Static*) outperforms the two Induced subnetworks (i.e., Induced-Dynamic and Induced-Static). However, we find this not to be the case. *In the context of cross-validation*, NetWalk-Dynamic, which performs the best among the above three unweighted NP-based subnetworks, has a tied performance to Induced-Dynamic in terms of recall and F-score but has a lower performance than Induced-Dynamic in terms of AUPR and precision (Fig. 2). Also, NetWalk-Dynamic has a lower performance than Induced-Static in terms of AUPR, recall, and F-score, but a marginally better performance than Induced-Static in terms of precision. Similarly, *in the context of cancer-related validation*, NetWalk-Dynamic has a lower performance than both Induced-Dynamic and Induced-Static (Fig. 4).

These results match our postulation that unweighted NetWalk-based subnetworks could be suboptimal because all of their PPIs are treated with equal importance (see *“Introduction”*). This is exactly why we propose to infer a weighted subnetwork using NetWalk in the first place. Indeed, when we look into whether the best of the weighted NP-based subnetworks, i.e., *w*NetWalk-Dynamic, outperforms all Induced subnetworks, we find this to be the case in terms of most of the considered evaluation measures, as follows.

*In the context of cross-validation*, *w*NetWalk-Dynamic, when analyzed via our proposed novel weighted dynamic features, outperforms all Induced subnetworks in terms of AUPR, recall, and F-score. In terms of precision, while *w*NetWalk-Dynamic is marginally (i.e., not statistically significant) inferior to Induced-Dynamic, it still outperforms Induced-Static. The superiority of *w*NetWalk-Dynamic is statistically significant when compared to Induced-Dynamic in terms of AUPR, recall, and F-score, and when compared to Induced-Static in terms of AUPR and F-score (Fig. 2) (adjusted *p*-values $< 0.05$).

**Fig. 3:**
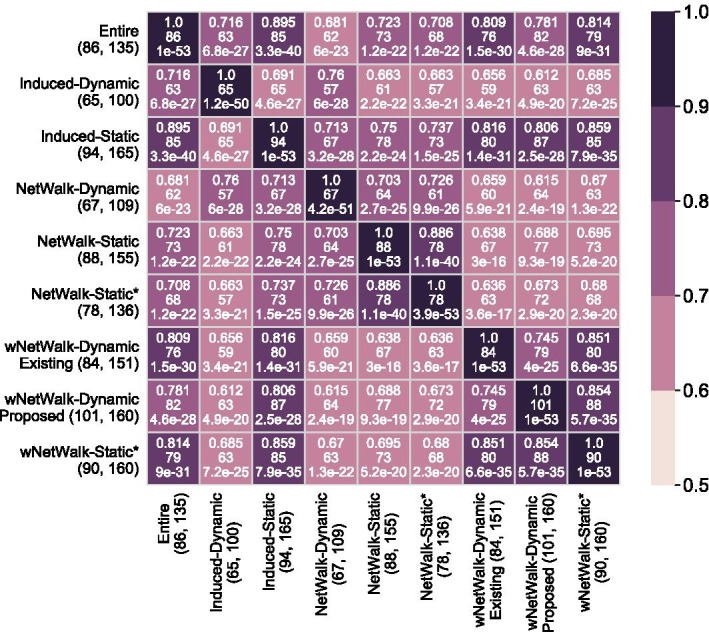
Overlaps (expressed as Jaccard indices) between true positive predictions of the eight considered (sub)networks, each under its best predictive model, when using the primary GenAge-based definition of aging- and non-aging-related gene labels, and when using HPRD. On the *x*- and *y*-axes, the numbers in the parentheses after the name of each subnetwork are the number of true positive predictions (i.e., known aging-related genes) and the number of total predictions. Within each table/matrix cell, the number on the first line is the Jaccard index of the overlap between the corresponding pair of subnetworks; the number on the second line is the raw overlap size, i.e., the actual count of true positives that are in the overlap between the two subnetworks; the number on the third line is the adjusted *p*-value of the overlap with respect to the hypergeometric test. The darker the color of a given cell, the larger the Jaccard index value, i.e., the higher the overlap. Note that we have two groups of results for *w*NetWalk-Dynamic, i.e., *w*NetWalk-Dynamic Existing and *w*NetWalk-Dynamic Proposed, which we already explained in the caption of Fig. 2. For results when using BioGRID instead of HPRD, see Additional file 1: Fig. S5

For all four cross-validation prediction accuracy measures, all (sub)networks perform statistically significantly better than the random approach (adjusted *p*-values $\le 0.032$). That is, each (sub)network predicts a majority (over 56%) of the genes that are labeled as aging-related (i.e., are known aging-related genes). So, we expect the pairwise overlaps of all (sub)networks’ true positives to be reasonably large. Indeed, we find that overlaps are statistically significantly high for all (sub)network pairs. The largest overlap over all (sub)network pairs is 89.5%, the smallest one is 61.2%, and the average one is 72.9% (Fig. 3). We note that even though our *w*NetWalk-Dynamic marginally underperforms Induced-Dynamic in terms of precision, *w*NetWalk-Dynamic not only correctly predicts 63 out of all 65 known aging-related genes that Induced-Dynamic predicts (i.e., 96.9% of them), but it also *correctly* predicts 38 known aging-related genes that Induced-Dynamic *does not* predict. Similarly, we examine pairwise overlaps of the (sub)networks’ predicted genes that are currently labeled as non-aging-related, i.e., of their *novel* aging-related gene predictions. We find that all of the pairwise overlaps are statistically significantly high. The largest overlap over all pairs is 62.2%, the smallest one is 17.9%, and the average one is 33.9% (Additional file 1: Fig. S2).

**Fig. 4:**
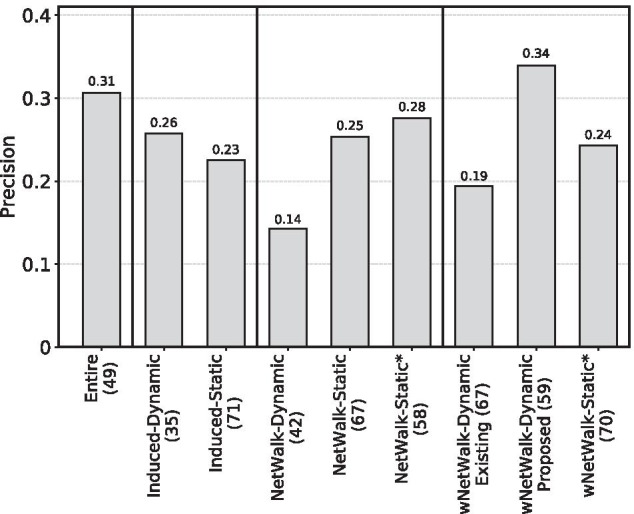
Cancer-related validation of the novel aging-related gene predictions for the eight considered (sub)networks, each under its best predictive model, when using the primary GenAge-based definition of aging- and non-aging-related gene labels, and when using HPRD. On the *x*-axis, the number in the parentheses after the name of each (sub)network is the number of the novel predictions. The precision score of a (sub)network is the percentage of novel predictions that are validated by the cancer-related data. Note again that we have two groups of results for *w*NetWalk-Dynamic, i.e., *w*NetWalk-Dynamic Existing and *w*NetWalk-Dynamic Proposed, which we already explained in the caption of Fig. 2. For results when using BioGRID instead of HPRD, see Additional file 1: Fig. S7

*In the context of cancer-related validation* (Fig. 4), we again find that *w*NetWalk-Dynamic (under our proposed novel weighted dynamic features) performs better than all Induced aging-specific subnetworks. That is, 34% of novel aging-related gene predictions (i.e., predicted genes currently not known to be aging-related) made by *w*NetWalk-Dynamic are present in the cancer-related data, while the best Induced aging-specific subnetwork (i.e., Induced-Dynamic) only gets 26% of novel predictions validated by the cancer-related data.

All of the above results signify that our hypothesis (1), i.e., that using an NP-based aging-specific subnetwork is better than using all Induced aging-specific subnetworks, holds with respect to almost all evaluation measures, i.e., AUPR, recall, F-score, pairwise prediction overlaps, as well as the cancer-related validation. This result stresses the significance of proposing our *w*NetWalk-Dynamic.

### Using dynamic versus static NP-based aging-specific subnetworks

Here, we evaluate our hypothesis (2): whether using the best of the *NP-based dynamic* aging-specific subnetwork will outperform all *NP-based static* aging-specific subnetworks. Because *w*NetWalk-Dynamic is superior to (unweighted) NetWalk-Dynamic, it is *w*NetWalk-Dynamic that we compare to the NP-based static subnetworks (i.e., NetWalk-Static, NetWalk-Static*, and *w*NetWalk-Static*).

*In the context of cross-validation*, we find that *w*NetWalk-Dynamic indeed outperforms all three static NP-based subnetworks in terms of all four prediction accuracy measures (Fig. 2). The superiority of *w*NetWalk-Dynamic is statistically significant when compared to NetWalk-Static* in terms of recall, and when compared to *w*NetWalk-Static* in terms of AUPR (adjusted *p*-values $< 0.05$), while all other superiorities of *w*NetWalk-Dynamic are non-significant. When we look into the pairwise prediction overlaps between the dynamic vs. static NP-based subnetworks (Fig. 3), we confirm *w*NetWalk-Dynamic’s superiority. Namely, it is *w*NetWalk-Dynamic that not only has the highest precision, but also has the highest number of predictions than any of the other NP-based subnetworks.

*In the context of cancer-related validation*, we again confirm the superiority of *w*NetWalk-Dynamic (Fig. 4). That is, 34% of the novel aging-related genes predicted by *w*NetWalk-Dynamic are present in the cancer-related data. At the same time, the best static NP-based subnetwork (i.e., NetWalk-Static*) only has 28% of its novel predictions present in the cancer-related data.

Therefore, hypothesis (2), i.e., that using an *NP-based dynamic* aging-specific subnetwork is better than using all *NP-based static* subnetworks, holds with respect to all evaluation measures. This result further stresses the significance of proposing our weighted dynamic NP-based aging-specific subnetwork.

### Using weighted versus unweighted dynamic NP-based aging-specific subnetworks

Here, we evaluate our hypothesis (3): whether using the *weighted dynamic* NP-based aging-specific subnetwork (i.e., *w*NetWalk-Dynamic) is better than using the *unweighted dynamic* NP-based aging-specific subnetwork (i.e., NetWalk-Dynamic). Indeed, we find this to be the case.

*In the context of cross-validation*, as shown in Fig. 2, *w*NetWalk-Dynamic outperforms NetWalk-Dynamic in terms of all four prediction accuracy measures. Of the four, the superiority is statistically significantly high (adjusted *p*-values $< 0.05$) in terms of recall and F-score. When we look into the pairwise prediction overlaps between *w*NetWalk-Dynamic and NetWalk-Dynamic, we find that *w*NetWalk-Dynamic not only correctly predicts 64 out of all 67 known aging-related genes that NetWalk-Dynamic predicts (i.e., 95.5% of them), but it also *correctly* predicts 37 known aging-related genes that NetWalk-Dynamic *does not* predict.

*In the cancer-related validation*, we again find *w*NetWalk-Dynamic to be better (Fig. 4). Specifically, among the novel aging-related genes (currently not known to be aging-related) predicted by *w*NetWalk-Dynamic and NetWalk-Dynamic, 34% and only 14%, respectively, are among the cancer-related genes.

Henceforth, hypothesis (3), i.e., that using *w*NetWalk-Dynamic is superior to using NetWalk-Dynamic, holds with respect to all evaluation measures. This result again stresses the importance of inferring our weighted NP-based dynamic aging-specific subnetwork.

### Using our proposed versus existing weighted dynamic features

Here, we evaluate our hypothesis (4): whether using *our proposed* weighted dynamic features will be better than using the *existing* weighted dynamic features. To validate our hypothesis (4), given *w*NetWalk-Dynamic, it would suffice for our best weighted dynamic feature to outperform the best existing weighted dynamic feature. So, given *w*NetWalk-Dynamic, we choose the best predictive model based on the considered existing weighted dynamic features and the best predictive model based on our proposed weighted dynamic features, (see *“Methods”* and Additional file 1: Section S2). Then, we compare the two chosen best models using cross-validation, prediction overlap, and cancer-related validation.

*In the context of cross-validation*, we find that our best proposed feature outperforms the best existing feature in terms of all four prediction accuracy measures. Of the four measures, the superiority is statistically significant in terms of AUPR, recall, and F-score (i.e., adjusted *p*-values $< 0.05$) (Fig. 2). When we look into the pairwise prediction overlaps between our best proposed feature and the best existing feature, we find that the best proposed feature not only correctly predicts 79 out of all 84 known aging-related genes that the best existing feature predicts (i.e., 94.0% of them), but it also *correctly* predicts 22 known aging-related genes that the best existing feature *does not* predict.

*In the context of cancer-related validation*, our best proposed feature again wins over the best existing feature. Specifically, 34% of the novel aging-related genes predicted by the former and only 19% of the novel aging-related genes predicted by the latter are present in the cancer-related data.

Therefore, our hypothesis (4), i.e., that using the best of our proposed weighted dynamic features is better than using all of the existing weighted dynamic features, holds with respect to all evaluation measures.

### Literature validation of novel aging-related gene predictions resulting from the best subnetwork, i.e., *w*NetWalk-Dynamic

Because *w*NetWalk-Dynamic (under the best of our proposed features) is the best subnetwork among all eight considered (sub)networks, those genes that are predicted by *w*NetWalk-Dynamic as aging-related but are currently not known to be linked to aging (i.e., that are novel aging-related gene predictions) are the most confident predictions for future experimental validation. There are 59 such novel predictions. Because it would be extremely time consuming to validate all of the 59 novel predictions, we aim to validate using literature search those novel predictions that are predicted *only by* *w*NetWalk-Dynamic but not by any other (sub)network. There are six such predictions, i.e., ATF1, GLI3, JUNB, NRP1, SMC1A, and SPI1. When we manually search for a direct (or indirect) link between these six genes and the aging process (or an aging-related disease) in PubMed articles, we manage to validate all six of them. Namely:

- *ATF1* was shown to be related to the pathogenesis of the Alzheimer’s disease [35], which is known to be highly related to aging [36].
- *GLI3* was shown to be linked to tumorigenicity of the colorectal cancer [37], which was shown to be mainly affecting the elderly [38].
- *JUNB*’s expression levels were shown to increase in all immune cells during the aging process [39].
- *NRP1* was shown to be overexpressed in various human carcinoma cell lines and primary tumors [40], which are the main causes of skin cancer that usually appeared in old people [41].
- The mutation of *SMC1A* was detected in colorectal cancer [42], which was shown to be mainly affecting the elderly [38].
- *SPI1* was identified as being upregulated in Alzheimer’s disease [43], which is known to be highly related to aging [36].

### Results when using our secondary aging-related gene label definition with respect to GTEx-DAG

All results reported so far are for when using the GenAge-based labels to define genes as aging- vs. non-aging-related, and when using HPRD. Here, we report results when using the GTEx-DAG-based labels while still using HPRD. The (sub)networks’ best predictive models when using GTEx-DAG are shown in Additional file 1: Table S2.

*In the context of cross-validation*, like for the GenAge-based labels, we do see that *w*NetWalk-Dynamic again performs the best among all tested eight (sub)networks in terms of all prediction accuracy measures. Unlike for the GenAge-based labels, we now also see that (unweighted) NetWalk-Dynamic outperforms both Induced subnetworks. These results further support the need for NP-based weighted dynamic aging-specific subnetworks (Additional file 1: Fig. S3).

However, in our previous studies [18], we already showed that for the two Induced subnetworks, prediction accuracy is not nearly as good under the GTEx-DAG-based gene labels as it is under the GenAge-based gene labels. Here, we confirm that the same holds for the NP-based (weighted and unweighted) subnetworks. Their prediction accuracy scores are not statistically significantly more accurate than the random approach (adjusted *p*-values $\ge 0.05$).

Because of the random-like performance of all (sub)networks with respect to GTEx-DAG, we do not perform analysis of pairwise prediction overlaps. Also, we do not make any novel predictions from the GTEx-DAG-based classifiers. Consequently, the cancer-related validation cannot be performed.

### Results for BioGRID-based (sub)networks

All results up to this point are for when using the HPRD entire context-unspecific network to infer all aging-specific subnetworks. In this subsection, we present results when using the BioGRID entire network instead. Note that in this case, we only use the GenAge-based aging- vs. non-aging-related gene labels. That is, we do not use GTEx-DAG-based labels when using BioGRID given their random-like performance when using HPRD (see above). The best predictive models for BioGRID-based (sub)networks (when using GenAge) are shown in Additional file 1: Table S3.

*In the context of cross-validation*, prediction accuracy scores for the BioGRID-based (sub)networks are statistically significantly higher than those of the random approach (adjusted *p*-values $< 0.05$). Similar to the HPRD-based subnetworks, for the BioGRID-based subnetworks, we see that *w*NetWalk-Dynamic performs the best among all tested eight (sub)networks in terms of all prediction accuracy measures. More importantly, *w*NetWalk-Dynamic statistically significantly (adjusted *p*-values $< 0.05$) outperforms most of the (sub)networks, for most of the considered performance measures. The only exceptions are: (1) Entire with respect to F-score and recall; (2) *w*NetWalk-Static with respect to all four prediction accuracy measures. These results further support the need for NP-based weighted dynamic aging-specific subnetworks (Additional file 1: Fig. S4). When we look into the actual prediction accuracy scores, we find that the scores are below 0.5 for all considered BioGRID-based (sub)networks (Additional file 1: Fig. S4), which overall is lower than for HPRD-based (sub)networks (Fig. 2). When we look into the raw prediction counts of aging-related genes (Additional file 1: Fig. S5 and S6), we find that overall there are more predictions but fewer true positives for BioGRID-based (sub)networks than for HPRD-based (sub)networks, which is why the performance of BioGRID is inferior to that of HPRD.

*In the context of cancer-related validation* (Additional file 1: Fig. S7), we again find that *w*NetWalk-Dynamic (under our proposed novel weighted dynamic features) performs better than all other (sub)networks. That is, 27% of novel aging-related gene predictions (i.e., predicted genes currently not known to be aging-related) made by *w*NetWalk-Dynamic are present in the cancer-related data, while the best unweighted (sub)network (i.e., Induced-Static) only gets 17% of novel predictions validated by the cancer-related data.

All of the above results signify that our four hypotheses hold not just when using HPRD but also when using BioGRID, because in both cases, using *w*NetWalk-Dynamic under our proposed weighted dynamic features performs the best. This again stresses the significance of proposing our *w*NetWalk-Dynamic and our weighted dynamic features, regardless of which entire human PPI network data we are using.

## Discussion

In this study, we have analyzed aging-specific subnetworks derived from one of two human PPI network data sets, namely HPRD and BioGRID. Some additional observations and implications of our results are as follows.

Recall that a predictive model consists of three individual components, i.e., a feature, a classifier, and a dimensionality reduction choice. We find that there is no clear pattern regarding which component of which model works well on which (sub)network (Table 3 and Additional file 1: Tables S2 and S3). This stresses the need for our comprehensive evaluation of running all of the different models, in order to give each (sub)network the best-case advantage. Also, it emphasizes the importance of a shift in the field of machine learning development of interpretable predictive models, in order to allow for understanding the effect of each model component on the prediction outcome. Furthermore, the superiority of our proposed weighted dynamic aging-specific subnetwork when using our proposed weighted dynamic features with respect to GenAge-based aging-related gene labels over all other (sub)networks in terms of almost all evaluation measures stress out the significance of our work.

The performance is inferior when using GTEx-DAG compared to when using GenAge (on HPRD-based subnetworks). This is surprising, for two reasons. First, genes in GTEx-DAG have been linked to aging directly in human, while those in GenAge have been linked to aging indirectly based on their sequence similarities to aging-related genes in model species. So, GTEx-DAG should better capture aspects of the aging process that are unique to human. Given this, and given that we have aimed to extract human aging-related knowledge from human PPI subnetworks, one would expect GTEx-DAG to perform better. However, it does not. This could be because GenAge may be more accurate than GTEx-DAG. In particular, GenAge has more rigorous selection criteria of aging-related genes than GTEx-DAG (see Section *“Data”* for more details). Second, the considered (sub)networks have been formed by integrating gene expression data with PPI data. Given that GTEx-DAG is also gene expression-based, while GenAge is sequence-based, one would expect GTEx-DAG to perform better. However, it does not. This could be because GTEx-DAG and GenAge are highly complementary (when considering HPRD, only 17 genes are labeled as aging-related in both), and because our predictive models may be better suited for the aging-related knowledge present in GenAge.

The performance is inferior when using the BioGRID network data compared to when using the HPRD network data (under GenAge-based aging-related label information). This could be because the aging-specific gene expression data used to infer aging-specific subnetworks originates from the same time period as HPRD, while the BioGRID data that we have used is much more recent. Newer aging-specific gene expression data, obtained using more modern technologies such as RNA-seq have become available, such as data from the Genotype-Tissue Expression project (GTEx) [44]. Analyzing BioGRID-based (sub)networks using the newer gene expression data may thus be promising and is the subject of our future work.

## Conclusions

In this study, we have proposed a novel NP-based weighted dynamic aging-specific subnetwork, along with novel weighted dynamic node features, which combined yield better prediction accuracy in supervised prediction of aging-related genes. We have used NP in hope of improving upon Induced that has been used traditionally. Indeed, this is what we observe.

Our findings are quantitatively robust to the choice of the entire context-unspecific PPI network used to infer aging-specific subnetworks. However, our findings are not quantitatively robust to the choice of ground truth aging-related data. Namely, our predictive models work better for sequence-based aging-specific expression data from model species than for gene expression-based aging-specific expression data from human. Development of predictive models that will be capable of capturing well human-unique aspects of the aging process (which do not exist in model species) is important.

Some ways to improve upon the current findings, including potentially on their robustness to data choice, could be as follows. First, in the current study, we use traditional machine learning where the user has to define a feature first and then “plug-in” this feature into an off-the-shelf classifier. However, lots of advancements have been made in deep learning on graphs, including graph convolutional networks (GCNs) for dynamic network analysis, in domains such as social or citation networks [45, 46]. So, it might be worth it to adopt or customize these existing GCNs to the computational biology domain and specifically to the task of studying the aging process. Second, the aging-specific gene expression data considered for subnetwork inference in the current study were curated a while ago. So, it might be worth it to adopt newer aging-specific gene expression data for this purpose, which is the subject of our future work.

We have studied human aging from PPI data. Nonetheless, our work can be applied to other types of biological networks, other species, or other biological processes, such as disease progression.

## Methods

### Data

#### The two considered entire context-unspecific PPI networks

In this study, we consider two entire context-unspecific PPI networks. The first considered unweighted entire context-unspecific PPI network in this study is obtained from the HPRD database [23]. We take the largest connected component that encompasses 8938 nodes and 35900 edges, and we denote it as Entire. The second considered unweighted entire context-unspecific PPI network in this study is obtained from the BioGRID database (i.e., version 4.4.197 released on April. 25th, 2021) [24]. We consider the largest connected component of human physical interactions from BioGRID, which encompasses 18,928 nodes and 484,146 edges. For each entire context-unspecific PPI network, we consider the following aging-specific subnetworks that are inferred from the given entire PPI network. We use HPRD entire network for illustration. All aging-specific subnetworks with respect to BioGRID are analogous to HPRD.

#### Seven considered aging-specific subnetworks

Other than Entire, we also consider seven aging-specific subnetworks that are inferred from Entire via two network inference methods, i.e., the Induced approach and the NP-based approach called NetWalk, as follows.

First, we use the dynamic aging-specific subnetwork inferred by Faisal and Milenković [4] using the Induced approach. Specifically, given human gene expression data at 37 ages spanning between 20 and 99 years [10], a dynamic aging-specific subnetwork consisting of 37 subnetwork snapshots, one snapshot per age, was constructed. Each subnetwork snapshot corresponded to the Induced subgraph of those genes that are significantly expressed (i.e., active) at the given age. Because the Induced approach does not map gene activities onto genes or PPIs (i.e., it does not assign weights to genes or PPIs), the 37 Induced subnetwork snapshots are unweighted. We refer to the resulting Induced unweighted dynamic aging-specific subnetwork, as Induced-Dynamic. Moreover, one of our key questions is whether aging-related genes can be predicted more accurately from a dynamic network than from a static network. So, we create an *unweighted* static counterpart for Induced-Dynamic, as follows. We aggregate (i.e., take the union of) the nodes and edges over all 37 snapshots such that a PPI is kept if it is present in any of the 37 subnetwork snapshots. We denote this static subnetwork as Induced-Static. This allows for a fair comparison between a dynamic subnetwork against its static counterpart, as the two contain the same nodes and edges.

Other than Induced-Dynamic, we also use an NP-based dynamic aging-specific subnetwork inferred in our previous work [19]. Key differences between the Induced approach and NP are discussed in Section *“Introduction”*. We extended two prominent NP approaches originally proposed for inference of a static cancer-specific subnetwork, i.e., NetWalk and HotNet2, to the problem of inferring a dynamic aging-specific subnetwork [19]. Specifically, given an NP approach, we created a dynamic aging-specific subnetwork with 37 age-specific subnetwork snapshots, as follows. Given Entire and expression levels for genes at a given age, we used the NP approach to assign age-specific weights to every PPI of Entire, such that the higher the weight of a PPI, the more important the PPI is. We did this for each of the 37 ages and obtained 37 age-specific weighted subnetwork snapshots. Finally, to obtain an unweighted dynamic aging-specific subnetwork, we only kept those “highly” weighted PPIs in each of the 37 age-specific weighted subnetwork snapshots, which resulted in the most relevant aging-specific dynamic subnetwork (i.e., the collection of the 37 age-specific subnetwork snapshots). See Newaz and Milenković [19] for details. We evaluated dynamic aging-specific subnetworks produced by NetWalk against those produced by HotNet2, and found the former to be better, i.e., to overlap more with aging-related ground truth data [19]. Consequently, in this paper, we only focus on the dynamic aging-specific subnetworks inferred using NetWalk, see Additional file 1: Section S3 for overview of NetWalk. For this NP approach, we considered two versions, referred to as option 1 and option 2 [19]. The key difference between these two versions lies in the different ways in which they process gene expression levels for each gene type (i.e., active or non-active genes) prior to propagating them through the network. Intuitively, the former assigns actual expression levels to only those genes that are significantly expressed (active) at a given age and “non-informative” (i.e., “dummy”) expression levels to all other genes in the entire network, while the latter assigns actual expression levels to both active and non-active genes. We have shown that the dynamic subnetwork inferred using option 2 (which was referred to as NetWalk* in the original publication [19]) overlapped better with the aging-related ground truth data than the dynamic subnetwork inferred using option 1 [19]. Hence, we use the dynamic subnetwork inferred using NetWalk option 2, which we refer to as NetWalk-Dynamic.

NetWalk-Dynamic was constructed by only keeping “highly” weighted PPIs. This means that a weight “threshold” was defined before the “highly” weighted PPIs were selected. In other words, those PPIs with weights less than a predefined threshold were removed. However, identifying such a threshold can be a time-consuming task. Additionally, we believe that keeping all of the PPIs and distinguishing between them using aging-specific weights is likely to capture more aging-specific information than only keeping a set of most “important” PPIs. That is, we aim to construct a dynamic aging-specific subnetwork such that (1) the aging-specific information can be distinguished via the PPIs’ weights and (2) a predefined weight “threshold” is not needed, as follows.

Given Entire and the gene expression data for 37 ages [10], we first use NetWalk (i.e., option 2) to obtain 37 weighted subnetwork snapshots in the same manner as done by Newaz and Milenković [19] (also described above). However, unlike Newaz and Milenković [19], we do not remove any of the PPIs from any of the 37 weighted subnetwork snapshots. Hence, each of the 37 snapshots has all of the PPIs from Entire. Second, to make the weights of PPIs comparable across different snapshots, we normalize the PPI weights over all 37 snapshots between the values of 0.01 and 1. Additionally, we believe that studying changes in the age-specific weights of PPIs across subsequent ages can capture more aging-specific information than just analyzing “raw” age-specific weights of PPIs. Hence, third, given the 37 snapshots with normalized PPI weights, we create 36 “differential” snapshots, as follows. Given two consecutive snapshots, i.e., *i* and $i+1$, we create a differential snapshot, such that, for a given PPI (e.g., *Y*), we define its differential weight ($Y^{wt}_{i,i+1}$) as the percentage change from its weight in snapshot *i* (i.e., $Y^{wt}_i$) to its weight in snapshot $i+1$ (i.e., $Y^{wt}_{i+1}$). Formally, we define the weight of the PPI in a differential snapshot ($i,i+1$) as $Y^{wt}_{i,i+1} = \frac{[Y^{wt}_{i+1} - Y^{wt}_i]\times 100}{[Y^{wt}_{i+1} + Y^{wt}_i]}$. Because there are 36 consecutive snapshots, we create 36 differential weighted subnetworks. We use the collection of 36 differential weighted subnetworks as the weighted NetWalk-Dynamic aging-specific subnetwork, which we refer to as *w*NetWalk-Dynamic. Note that because the differential edge weights are percentages of weight changes between two consecutive snapshots, the maximum and minimum possible differential edge weights are -100 and 100, respectively.

Hence, we consider two versions of NetWalk-based dynamic aging-specific subnetworks, i.e., the *unweighted* dynamic aging-specific subnetwork resulting from thresholding (NetWalk-Dynamic), and the *weighted* dynamic aging-specific network without thresholding (*w*NetWalk-Dynamic). Additionally, we consider a counterpart of *w*NetWalk-Dynamic, which we obtain as follows. We follow the same procedure that we do for *w*NetWalk-Dynamic up to the step where we obtain 37 snapshots with normalized edge weights. We consider this weighted dynamic subnetwork as the “non-differential” counterpart of *w*NetWalk.

We consider this “non-differential” counterpart of *w*NetWalk because of the following reasons. *w*NetWalk-Dynamic is a “differential” weighted subnetwork, where the weight of an edge captures a change between two consecutive snapshots. However, the considered existing weighted dynamic features were originally not defined for a differential weighted dynamic subnetwork. To give the existing weighted dynamic features the best-case advantage, we evaluate them not only on *w*NetWalk-Dynamic but also on the non-differential counterpart of *w*NetWalk-Dynamic. Because we find that the existing weighted dynamic features perform better with respect to the non-differential counterpart of *w*NetWalk-Dynamic than with respect to *w*NetWalk-Dynamic, we only report results of the existing weighted features based on the non-differential counterpart of *w*NetWalk-Dynamic.

Similar to Induced-Dynamic, we also need static counterparts for the dynamic NP-based subnetworks. We consider two versions of static subnetworks, i.e., static and static*. Because the first version is meant for unweighted dynamic subnetworks, we create the static counterpart only for NetWalk-Dynamic, which we denote as NetWalk-Static. On the other hand, because NP was originally proposed for static context-specific subnetwork, for an NP-based dynamic subnetwork, we use NP to infer another static subnetwork counterpart, i.e., static*, as follows. We first aggregate gene expression data over all ages (so that a node is considered active if it is active at any one or more of the 37 ages) and then integrate the aggregated gene expression-based activity data with Entire using NetWalk. The resulting static aging-specific subnetwork (without thresholding) is the counterpart of *w*NetWalk-Dynamic, and is denoted as wNetWalk-Static*. Then, to create the static counterpart of NetWalk-Dynamic, given *w*NetWalk-Static*, we choose the edge weight threshold that results in a static subnetwork matching the number of edges in NetWalk-Static, and we denote such static subnetwork as NetWalk-Static*. This way, the two versions of the static subnetworks, i.e., NetWalk-Static and NetWalk-Static*, are fairly comparable to each other.

In total, we analyze Entire which is unweighted, two unweighted Induced subnetworks, three unweighted NP-based subnetworks, and two weighted NP-based subnetworks. That is, we consider $1 + 2 + 3 + 2 = 8$ (sub)networks for each of the HPRD and BioGRID entire context-unspecific networks (Table 4).

**Table 4:** The sizes of the eight considered HPRD-based (sub)networks

Entire	Induced-Dynamic	Induced-Static	NetWalk-Dynamic
8,938; 35,900	4,696; 15,062	6,371; 22,975	3,749; 9,617
NetWalk-Static	NetWalk-Static*	*w*NetWalk-Dynamic	*w*NetWalk-Static*
4,973; 14,509	5,065; 14,509	8,938; 35,900	8,938; 35,900

The size of a dynamic subnetwork is the average over its 37 (or 36, depending on whether the considered dynamic subnetwork has 37 or 36 snapshots) subnetwork snapshots. The two numbers delimited by “;” are node and edge counts of the corresponding (sub)network. The sizes of the eight considered BioGRID-based (sub)networks are in Additional file 1: Table S4

#### Six considered aging-related ground truth data

The purpose of using the following data is described in the next section.

- GenAge [28] is a highly trusted source of human aging-related genes, most of which are sequence orthologs of experimentally validated aging-related genes in model organisms. In particular, a gene’s homolog in human is selected to be aging-related if the following criteria are met. (1) The gene’s influence in model organism’s aging process is experimentally validated; (2) There are multiple literature suggesting that the human homolog is implicated in multiple pathologies that are related to the aging process; (3) There is phenotypical information of mutations that the human homolog is involved; (4) The gene has effects on gene’s product(s) in mammals. Of all 307 human genes from GenAge, 185 are present in all eight considered HPRD-based (sub)networks. Of all 307 human genes from GenAge, 227 are present in all eight considered BioGRID-based (sub)networks. Henceforth, we denote the set of 185 HRPD-related or the set of 227 BioGRID-related GenAge genes as *GenAge*.
- GTEx [8] contains 863 genes whose expressions decrease with age (i.e., are *down-regulated*). Of which 347 are present in all eight considered HPRD-based (sub)networks and 691 are present in all eight considered BioGRID-based (sub)networks. Henceforth, we denote the set of 347 of HPRD-related or the set of 691 BioGRID-related GTEx down-regulated genes as *GTEx-DAG*.
- GTEx [8] contains 710 genes whose expressions increase with age (i.e., are *up-regulated*). Of which 161 are resent in all eight considered HPRD-based (sub)networks and 391 are resent in all eight considered BioGRID-based (sub)networks. Henceforth, we denote the set of 161 of HPRD-related or the set of 691 BioGRID-related GTEx up-regulated genes as *GTEx-UAG*.
- Lu et al. [9] identified 442 genes whose expressions vary with age, of which 265 are present in all eight considered HPRD-based (sub)networks and 306 are present in all eight considered BioGRID-based (sub)networks.
- Berchtold et al. [10] identified 8,277 genes whose expressions vary with age, of which 2,312 are present in all eight considered HPRD-based (sub)networks and 2,987 are present in in all eight considered BioGRID-based (sub)networks.
- Simpson et al. [12] identified 2,911 genes whose expressions vary across different stages of Alzheimer’s disease, of which 930 are present in all eight considered HPRD-based (sub)networks and 1,186 are present in all eight considered BioGRID-based (sub)networks.

#### Two considered definitions of (non-)aging-related gene labels

For supervised classification (discussed in the following sections), we need a set of genes labeled as aging-related (positive class) and a set of genes labeled as non-aging-related (negative class). Because GenAge is considered to be one of the most confident aging-related ground truth data sources, our primary label definition is with respect to GenAge. Human genes in GenAge are sequence orthologs of aging-related genes in model organisms. However, human aging is a complex process that has unique biological aspects compared to the aging process in a model species [34], which might be missed by GenAge. So, to hopefully capture the human-unique aspects, we separately consider a secondary label definition with respect to GTEx [8], which was obtained by studying aging directly in human. Specifically, of the entire GTEx, we focus on GTEx-DAG, because we analyze PPI data, and because genes in GTEx-DAG were shown to be relevant for PPIs, unlike genes in GTEx-UAG [8]. The two label definitions, i.e., based on either GenAge or GTEx-DAG, are as follows.

*Primary definition with respect to GenAge* We label as *aging-related* those 185 genes from GenAge that exist in all eight considered HPRD-based (sub)networks. Because we want our non-aging-related gene set to be as confident as possible, we label as *non-aging-related* those genes that exist in all eight considered (sub)networks but not in any of the six considered aging-related ground truth data; there are 1,485 such genes. We intentionally use the same 185-gene positive class and the same 1,485-gene negative class for classification in all eight HPRD-based (sub)networks. This allows us to fairly compare classification accuracy (i.e., the accuracy of aging-related gene prediction) across all (sub)networks. Similarly, we define GenAge-based aging and non-aging labels with respect to BioGRID, which results in 227 aging-related genes and 6,575 non-aging-related genes.

*Secondary definition with respect to GTEx-DAG* We label as *aging-related* those 347 genes from GTEx-DAG that exist in all eight (sub)networks. We label as *non-aging-related* those 1,485 genes that exist in all eight (sub)networks but not in any of the six considered aging-related ground truth data. Note that we use the GenAge definition separately from the GTEx-DAG definition, i.e., we perform classification for each of the two individually. This is because the two are very different (sequence- vs. expression-based) data that possibly capture different aspects of the aging process. Indeed, of the 185 GenAge-based and 347 GTEx-based aging-related genes, only 17 genes are common to the two gene sets.

#### Cancer-related genes

Vogelstein et al. [47] identified 138 mutation-related cancer driver genes, i.e., genes whose intragenic mutations contribute to cancer. Sondka et al. [48] identified 723 cancer driver genes by manually curating cancer-related genes from COSMIC to determine the presence of somatic mutation patterns in cancer. We combine these two sets of cancer-related genes, and obtain 728 unique cancer-related genes. Of these 728 genes, 366 are present in each of the eight HPRD-based (sub)networks analyzed in this study. Similarly, 524 are present in each of the eight BioGRID-based (sub)networks analyzed in this study. Henceforth, we denote the 366 HPRD-related or the 524 BioGRID-related cancer genes as *cancer-related genes*. Of the 366 HPRD-related cancer genes, 49 are aging-related and 87 are non-aging-related according to the primary GenAge-based definition. Of the 524 BioGRID-related cancer genes, 57 are aging-related and 176 are non-aging-related according to the primary GenAge-based definition. Also, among the 366 HPRD-related cancer genes, 15 are aging-related and 87 are non-aging-related according to the secondary GTEx-DAG-based definition.

### Predictive models: node features and classifiers

For existing node features and classifiers, please see Additional file 1: Section S1.1.

#### The proposed weighted features

Different types of network neighborhoods of a node typically capture different characteristics of the node’s network position. Hence, given a weighted network, we propose to extract features of a node using four network neighborhood types. That is, for a given node *v*, we extract its features using (1) edges connecting *v* to its direct (one-hop) neighbors, (2) edges among one-hop neighbors of *v*, (3) edges between one-hop neighbors of *v* and two-hop neighbors of *v*, and (4) edges among two-hop neighbors of *v*. We characterize a neighborhood type of a node by measuring the distribution of weights of the corresponding edges. Specifically, given *W* different edge weights in the dynamic weighted subnetwork, we count the number of times each of the *W* edge weights occur in the given neighborhood type. In more detail, given a dynamic weighted subnetwork (i.e., *w*NetWalk-Dynamic) with *N* snapshots, we create a feature vector of a node using three different approaches, as follows.

- Approach 1: for a given neighborhood type, for a given snapshot, for a given node, we compute counts of each of the *W* edge weights among its neighbors, resulting in a feature vector of size *W*. Then, we concatenate this feature vector from each of the *N* snapshots to get a feature vector of length $W\times N$. This results in four feature vectors, one corresponding to each of the four neighborhood types. Additionally, we define a feature vector that incorporates all neighborhood types, as follows. For each snapshot, we concatenate the size *W* feature vectors from each of the four neighborhood types to get a feature vector of size $W\times 4$. Then, we combine these concatenated feature vectors from each of the *N* snapshots into a single feature vector of size $W\times 4 \times N$. So, in total, we create five feature vectors for each node.
- Approach 2: for a given neighborhood type, for a given node, we measure the Pearson correlation of weight counts between each pair of *N* snapshots, resulting in a correlation matrix of size $N \times N$, where we take its upper triangular matrix as a feature vector of size $N\times (N-1)/2$. We do this for each of the four neighborhood types separately, which results in four feature vectors. Additionally, to incorporate all four neighborhood types in a single feature vector, we concatenate the feature vectors corresponding to the four neighborhood types to obtain another feature vector of size $4\times {N\times (N-1)/2}$. So, in total, we create five features for each node.
- Approach 3: for a given neighborhood type, for a given snapshot, for a given node, we measure the deviation of the distribution of the edge weights in the neighborhood from the distribution of edge weights in the whole weighted dynamic subnetwork. We use the Cramér-von Mises statistic [49] to measure the deviation between the two distributions, resulting in a “distance number” that quantifies the distance between the two distributions. Then, given *N* snapshots, we combine the *N* distance numbers to obtain a single feature vector of size *N*. We do this for each of the four neighborhood types separately, which results in four feature vectors. Additionally, to incorporate all four neighborhood types in a single feature vector, we concatenate the feature vectors corresponding to the four neighborhood types to obtain another feature vector of size $N \times 4$. So, in total, we create five feature vectors for each node.

The above procedure results in 15 weighted dynamic feature vectors for a node. In addition to the above way of characterizing the distribution of weights, where we count the occurrences of “raw” edge weights, we characterize the distribution of “binned” weights, where we bin the *W* edge weights into 200 bins, as follows. We first divide the interval between the smallest possible weight (i.e., $-100$) and the largest possible weight (i.e., 100) in a weighted dynamic subnetwork into equal-sized bins of size of 1, and then we assign all of the weights in the weighted dynamic subnetwork to their corresponding bins. Finally, we count how many edge weights belong to a given bin, to obtain the corresponding binned weight distributions. Finally, we use the same three approaches as above, i.e., approach 1, approach 2, and approach 3, to create 15 additional weighted dynamic feature vectors. Hence, in total, we create 30 weighted dynamic feature vectors, 15 using raw (“nobin”) weights, and 15 using binned (“bin”) weights.

Given aging-related ground truth data, we compare the 30 weighted dynamic features with respect to their ability to predict aging-related genes, in order to select the best weighted dynamic feature. Given the HPRD-based *w*NetWalk-Dynamic, for the primary definition of aging- and non-aging-related genes using GenAge data, the best weighted dynamic feature vector corresponds to the case when we use distributions of raw weights (i.e., approach 1 above) of the second neighborhood type of a node. We call this feature vector “Diff-nobin-2”. Given the HPRD-based *w*NetWalk-Dynamic, for the secondary definition of aging- and non-aging-related genes using GTEx-DAG data, the best weighted dynamic feature vector corresponds to the case when we use distributions of binned weights (i.e., approach 1 above) of the first neighborhood type of a node. We call this feature vector “Diff-bin-1”. Given the BioGRID-based *w*NetWalk-Dynamic, for the primary definition of aging- and non-aging-related genes using GenAge data, the best weighted dynamic feature vector corresponds to the case when we use Pearson correlation of raw weights (i.e., approach 2 above) of the second neighborhood type of a node. We call this feature vector “Diff-nobin-cor-2”.

To fairly compare *w*NetWalk-Dynamic to its static counterpart (i.e., *w*NetWalk-Static*), we create the corresponding static version of the best weighted dynamic feature, as follows. Given *w*NetWalk-Static*, we use the same approach to extract a weighted static feature that we use to create the best selected weighted dynamic feature. We identify the corresponding static features as “Static-nobin-2”, “Static-bin-1” and “Static-nobin-2” that correspond to Diff-nobin-2, Diff-bin-1, and Diff-nobin-cor-2, respectively.

## Supplementary Information

**Additional file 1**. Supporting methodological details of existing node features, evaluation framework, and additional results for HPRD-based (sub)networks and BioGRID-based (sub)networks.

## Data Availability

All data generated and analyzed during this study are available in the cone repository, https://nd.edu/~cone/WeightedSuperAge. The expression data [10] for the inference of our aging-specific subnetworks is GSE11882.

## Abbreviations

ADEx2011: Alzheimer’s disease expression 2011—a set of genes identified in 2011 as having their expressions change across different stages of Alzheimer’s disease
AUPR: Area under the precision-recall curve
BetwC-wt: Weighted betweenness centrality
BEx2004: Brain expression 2004—a set of genes identified in 2004 as having their expressions in a brain tissue change with age
BEx2008: Brain expression 2008—a set of genes identified in 2008 as having their expressions in a brain tissue change with age
BioGRID: Biological general repository for interaction datasets
CentraMV: Centrality mean and variation
CloseC-wt: Weighted closeness centrality
ClusC-wt: Weighted clustering coefficient
DegC: Degree centrality
DegC-wt: Weighted degree centrality
DGDV: Dynamic graphlet degree vector
ECC: Eccentricity centrality
EigenC-wt: Weighted eigen vector centrality
FDR: False discovery rate
FN: False negative
FP: False positive
GCN: Graph convolutional networks
GDC: Graphlet degree centrality
GenAge: The ageing gene database
GoT: Graphlet orbit transitions
GTEx-DAG: Genotype-tissue expression project—downregulated aging-related genes
GTEx-UAG: Genotype-tissue expression project—upregulated aging-related genes
HPRD: Human protein reference database
HuRI: Human reference interactome
KC: *k*-coreness centrality
LR: Logistic regression
*m*BPIs: Top *m* most important binary protein interaction attributes
NB: Naive Bayes
NP: Network propagation
PCA: Principal component analysis
PPI: Protein–protein interaction
SGDV: Static graphlet degree vector
SVM: Support vector machine
SVM-rbf: Support vector machine with a non-linear radial basis function kernel
TN: True negative
TP: True positive
*t*SNE: *t*-distributed stochastic neighbor embedding
UniNet: Union of 11 network centrality measures

## Footnotes

1 https://www.cdc.gov/nchs/nvss/vsrr/COVID19.
