# Supplementary material for: Improved supervised prediction of aging-related genes via weighted dynamic network analysis
Source: BMC Bioinformatics. 2021 Oct 25;22:520. doi: 10.1186/s12859-021-04439-3 (PMC8543111; doi:10.1186/s12859-021-04439-3)
Supplement: Supplementary file 1 — Additional file 1. Supporting methodological details of existing node features, evaluation framework, and additional results for HPRD-based (sub)networks and BioGRID-based (sub)networks. [file 12859_2021_4439_MOESM1_ESM.pdf]

Supplementary material for: Improved supervised prediction of aging-related genes via weighted dynamic network analysis

I Supplementary Sections

S1 Methods

S1.1 Predictive models: existing node features and classifiers

S1.1.1 The considered existing unweighted dynamic features

- DGDV, dynamic graphlet degree vector [1], counts how many times a node participates in each considered dynamic graphlet. Dynamic graphlets are an extension of static graphlets (small subgraphs on up to n nodes) to the dynamic context, where the temporal order in which events occur in a dynamic network is added onto edges of a static graphlet. As in the original DGDV paper [1], we use up to 4-node and 6-event graphlets, resulting in a 3,727-dimensional DGDV node feature. We could not run DGDV on the BioGRID-based Induced-Dynamic and NetWalk-Dynamic, as the large sizes of these two networks make this computationally prohibitive.

- GoT, graphlet orbit transitions [2], of a node counts, for every pair of considered graphlets, how many times one graphlet changes into the other. As in the original GoT publication [2], and for a fair comparison with DGDV, we use 4-node graphlets, resulting in a 121-dimensional GoT node feature. We could not run GoT on the BioGRID-based Induced-Dynamic and NetWalk-Dynamic, as the large sizes of these two networks make this computationally prohibitive.

- GDC, graphlet degree centrality [3], is defined for a node in a static network (as discussed below). We use GDC to compute, for each node v, v's centrality in each of the 37 snapshots of a dynamic subnetwork. Then, we combine v's 37 GDC values into its 37-dimensional dynamic GDC feature. We do the same for all other considered centrality features (listed below), each of which results in a 37-dimensional node feature. Going back to the definition of GDC in a static network: it ranks a node v as central if v participates in many graphlets or in complex (large or dense) graphlets (or both).

  For GDC, we use the code by Faisal and Milenković [4], which considers up to 5-node graphlets without an option to customize the graphlet size.

- ECC, eccentricity centrality [4], of a node v calculates distance (i.e., the shortest path length) between v and each other node in the network, finds a node u that is the most distant to v, and computes the reciprocal of the distance between u and v.

- KC, k-coreness [4], of a node v is the size of the largest network core to which v belongs. A network core is a subgraph in which each node is connected to at least k other nodes. When v belongs to a k-core, it also belongs to 1-core, 2-core, 3-core, ..., (k-1)-core. Then, KC of v is the size of its largest k-core.

- DegC, degree centrality [4], of a node v measures how many other nodes in the network v is connected to.

- CentraMV, centrality mean and variation [5, 6], of a node v measures, for each of the four considered centrality-based features (GDC, ECC, KC, and DegC), two quantities: the mean and variation over v's 37 centrality values. These two quantities for each of the four centrality-based features combined form an 8-dimensional CentraMV node feature.

S1.1.2 The considered existing unweighted static features

- SGDV, static graphlet degree vector [7], a static counterpart of DGDV, counts how many times a node participates in each considered static graphlet. Just as for DGDV and GoT, we consider up to 4-node static graphlets. This results in a 15-dimensional SGDV node feature. We have tested up to 5-node SGDV in this study, but this has not yielded an improvement (results not shown). So, for simplicity, we just report results for up to 4-node SGDV.

- UniNet [8] aggregates a number of node centralities (DegC, ECC, KC, average shortest path, betweenness, closeness, clustering coefficient, neighborhood connectivity, radiality, stress, and topological coefficient) into an 11-dimensional node feature.

- mBPIs [9] works as follows. First, we rank the genes (i.e., nodes) in the network based on their degrees from high to low, where nodes with the same degree are ranked the same. Then, we take the m highest-degree nodes from the ranked list of nodes. Then, for a node v, its feature has m dimensions corresponding to the m nodes, where each dimension d of the node v's feature has a value of 1 if v interacts with the top m highest-degree node d, or it has a value of 0 if v does not interact with d. Just as [5, 6], and as suggested in the original mBPIs publication, we use $m = 30$ in this paper. This results in a 30-dimensional mBPIs node feature. Two exceptions are for NetWalk-Static and NetWalk-Static*, where we use mBPIs node features with a dimension of 31 instead of 30. This is because, in each of the subnetworks, two genes are tied at rank 30, and so we include both genes in the set of top 30 highest-degree nodes.

S1.1.3 The considered existing weighted dynamic features

- DegC-wt [10], weighted degree centrality (or strength) is defined as the sum of the weights of edges connecting a node to its direct neighbors.

- ClusC-wt [11], weighted clustering coefficient captures how well connected are the direct neighbors of a node, while also taking into account weights of the edges connecting the node to its direct neighbors and the edges among direct neighbors. Several definitions of weighted clustering coefficient of a node has been defined. Because it has been shown that there is no best weighted clustering coefficient definition [12], given a node i, we use the following definition by Barrat *et al.* [11]: $C(i) = \frac{1}{s_i \cdot (d_i - 1)} \sum_{j,k} \frac{W_{ij} + W_{ik}}{2}$. Here, W_{ij} and W_{ik} are the weights of edges connecting the node i to nodes j and k, respectively, where j and k should be connected to each other, s_i is the strength of the node i, and d_i is the degree of the node i.

- CloseC-wt [10], weighted closeness centrality measures how close a node is on average to all other network nodes, by computing the inverse of the sum of weighted shortest paths from the given node to all of the other network nodes. Here, a weighted shortest path between any two nodes is the path whose sum of the inverse of the corresponding edge weights is minimum.

- BetwC-wt [10], weighted betweenness centrality of a node measures the fraction of all weighted shortest paths in a network that pass through the given node. Here, a weighted shortest path is defined in the same way as above.

- EigenC-wt [13], weighted eigenvector centrality measures the importance of a node based on how often the node is connected to other nodes of higher weighted degrees. More formally, weighted eigenvector centralities of nodes in a network correspond to the values in the first eigenvector of the weighted adjacency matrix of the corresponding network.

S1.1.4 Two considered feature dimensionality reduction choices

A common problem in supervised classification is overfitting, which often happens due to high dimensions of features used in the classification task [14]. Some of our considered features, including DGDV, GoT, and our proposed weighted dynamic features, have high dimensions. So, we apply linear feature dimensionality reduction (i.e., PCA) to all of our features. We are aware of more recent proposed nonlinear dimensionality reduction techniques, such as t-distributed stochastic neighbor embedding (tSNE) [15]. We tested tSNE under six perplexity parameters (5, 13, 21, 30, 40, 50) in our previous work [6]. However, we did not find any such case in which tSNE performs the best in terms of prediction accuracy, which is why we no longer test tSNE

in this study. Hence, for each feature, we consider *(i)* the full feature (i.e., no dimensionality reduction) and *(ii)* linear PCA that considers as few principal components as needed to account for at least 90% of variation in the data corresponding to the given feature. We perform feature dimensionality reduction during cross-validation, same as our previous study [6].

S1.1.5 Three considered classifiers

Among the nine classifiers that we considered in our previous studies [5, 6], we select three classifiers that perform better than the other classifiers in supervised prediction of aging-related genes, i.e., LR [16], SVM-rbf [17], and NB [18]. Briefly, LR is a classification algorithm that uses a logistic function to model a binary dependent variable. It usually performs well when the features are approximately linear and targets are linearly separable. NB is a family of simple "probabilistic classifiers" that applies Bayes's theorem with an assumption that the features are independent of each other. We use Gaussian NB in our study. SVM outputs an optimal hyperplane by using a hinge loss function to categorize objects. It can be used with multiple kernel functions depending on whether the objects are linearly separable or not. We use a non-linear kernel (radial basis function (SVM-rbf)). All classifiers are implemented using a Python library scikit-learn (version 0.23.1) [19].

S1.2 Evaluation

S1.2.1 Predictive models

For each definition of aging- and non-aging-related gene labels, for each of the eight (sub)networks, we develop a set of *predictive models*, each derived from unique combinations of a node feature, a dimensionality reduction choice, and a classifier. In particular, we consider 46 node features, where seven are unweighted dynamic, three are unweighted static, 35 are weighted dynamic, and one is weighted static. For each feature, we consider two dimensionality reduction choices (i.e., PCA vs no dimensionality reduction). Then for each feature version (i.e., PCA reduced version and full version), we consider three classifiers. Note that for weighted dynamic features, we only perform PCA on the best existing and the best proposed weighted dynamic feature because performing PCA on every weighted dynamic feature is computationally expensive due to their high dimensionalities. If some of the weighted features perform even better under PCA compared to no dimensionality reduction, it would further strengthen our hypotheses. We summarize the number of predictive models (i.e., 234) in Supplementary Table S1. Note that these 234 models are just for the GenAge-based definition of aging- and non-aging-related gene labels. The number is the same for the GTEx-based definition. So, over the entire study, we develop and evaluate a total of 468 predictive models. We present this number to give an idea to the reader of how comprehensive our study is.

S1.2.2 Hyperparameter training, cross-validation and prediction of aging-related genes

We run each predictive model under a systematic 5-fold cross-validation framework [6] via two steps, *(i)* hyperparameter tuning and *(ii)* model training and testing.

Specifically, we first randomly split the 185 aging- and 1485 non-aging-related genes into five equal-size subsets and perform 5-fold cross-validation. For each fold, one subset of the aging- and non-aging-related genes is treated as the testing data, and the remaining four subsets are treated as the training data. We denote them as model testing and model training data. Unlike our previous study [5, 6], where we used the default hyperparameter values for the considered classifiers, in our present study, we use each model training data to perform hyperparameter tuning, in order to give each model the best-case advantage. To do this, we further perform 5-fold cross-validation. That is, given a model training data, we randomly split the aging- and non-aging-related genes into five equal-sized subsets. A subset of the aging- and non-aging-related genes is treated as the testing data, and the remaining four subsets are treated as the training data. We denote them as tuning testing and tuning training data because they are generated for hyperparameter tuning. We train a given model via different hyperparameters on tuning training data and test it on tuning testing data. We quantify the performance using the average AUPR and select the "best" set of hyperparameter(s) that yields the highest average AUPR over the 5 folds. Note that this is only for one fold of the model training and model testing data. Because we have five folds, we have five sets of hyperparameter(s) (each set corresponding to a fold) for a given predictive model. We perform hyperparameter tuning for both LR and SVM-rbf. There is no hyperparameter for NB, so we use the default setting. The hyperparameter for LR is

the regularization strength for which we select 10 values between 2^{-8} to 2^8. There are two hyperparameters for SVM-rbf, i.e., gamma and regularization strength. We select 10 values between 2^{-8} to 2^8 for each of the two hyperparameters, which results in $10 \times 10 = 100$ sets of hyperparameters via grid search.

Given the best predictive model, we test the trained model on the testing data. Given a testing set, the output is a ranked list of the genes based on their predicted likelihood of being aging-related. If multiple genes have the same probability of being aging-related genes, their ranks are the same. For example, probabilities of $1.0, 0.95, 0.95, 0.9$ are ranked as $1, 2, 2, 4$. Then, to make aging-related gene predictions using a model, a threshold k needs to be selected so that those genes whose likelihoods of being aging-related are above the threshold are predicted as aging-related. We vary the number of predictions k from 1 to $\lceil (185+1485)/5 \rceil = 334$, in the increments of 1.

S1.2.3 Evaluation measures

For each fold, for predicted genes at each prediction threshold k, we use their actual aging- and non-aging-related labels to compute the numbers of true positives (TPs), false positives (FPs), and false negatives (FNs). A gene is considered to be TP if it is predicted to be aging-related and is also labeled as aging-related. A gene is considered to be FP if it is predicted to be aging-related but is labeled as non-aging-related. A gene is considered to be FN if it is *not* predicted to be aging-related but is labeled as aging-related.

Given TPs, FPs, and FNs for each prediction threshold for each of the five folds, we evaluate accuracy of a predictive model using average precision, average recall, average F-score, and average AUPR over five folds, where, precision is # of TPs/(# of TPs + # of FPs), recall is # of TPs/(# of TPs + # of FNs), F-score is the harmonic mean of precision and recall, and AUPR is the area under the precision recall curve. Specifically, we compute these measures as follows.

Given a predictive model, we compute average precision, average recall, and average F-score using the following procedure. First, we select a single prediction threshold $k1$, which is the same for all of the five folds. Second, for each fold, we compute fold-specific precision, recall, and F-score at $k1$. Third, we take the average of fold-specific precision, recall, and F-score values at $k1$ over all of the five folds to obtain the average precision, average recall, and average F-score, respectively. We select the prediction threshold $k1$ as follows. For each prediction threshold k, we combine the predictions from each fold into a single list, and calculate the precision, recall, and F-score. We believe that for potential wet lab validation of predictions, precision should be favored over recall, as long as recall is not too low [5, 6]. Because maximizing F-score is exactly what ensures that recall is not too low, i.e., that both precision and recall are reasonably high, we select the prediction threshold $k1$ where F-score is maximized, while at the same time precision is at least as large as recall. As such, the number of aging-related gene predictions for a given network is $5 \times k1$.

Given a predictive model, we compute the average AUPR as follows. First, given precision and recall values for each prediction threshold k in a given fold, we compute the fold-specific AUPR. Then, we take average of the fold-specific AUPRs over the five folds to obtain a single average AUPR value.

Note that after we compute the above average precision, average recall, average F-score, and average AUPR values, we round each of them to the second decimal place to avoid marginal superiority of one predictive model to another. Specifically, considering accuracy values rounded to the second decimal place ensures that we only consider a predictive model to have higher accuracy than another if the former is at least 0.5% more accurate than the latter.

S1.2.4 Choice of the best predictive model for each (sub)network

For each of the eight (sub)networks, we rank the predictive models based on their average AUPR, average F-score, average precision, and average recall. Then, we select the "best" predictive model with the highest rank, i.e., the one that maximizes the average AUPR, to give it the best-case advantage. Then, we report average AUPR, average precision, average recall, and average F-score of the selected predictive model.

S1.2.5 Statistical significance of a predictive model's accuracy

First, we compare the accuracy of each selected predictive model against the accuracy of a random approach. The latter works as follows. For a given testing data (i.e., cross-validation fold) and each prediction threshold k, we randomly select k testing genes and predict them as aging-related. Then, we repeat this for each of the five folds. To account for randomness, we run these two steps 30 times, which means that we perform

$5 \times 30 = 150$ random aging-related gene predictions. We report the accuracy scores (AUPR, precision, recall, and F-score) of the random approach as the average over the 150 runs. A predictive model is good only if its accuracy is statistically significantly higher than that of a random approach.

Second, we compare pairs of the selected predictive models (plus the random approach), to determine if one model's predicted gene set is statistically significantly more accurate than another model's predicted gene set. We do this by comparing the two models' five accuracy scores corresponding to the five folds via the paired Wilcoxon signed-rank test [20]. Because we perform this test for multiple pairs of models, we apply the false discovery rate (FDR) correction [21] to adjust the p-values. We choose 0.05 as the significance level (i.e., one model is statistically significantly better than another if the adjusted p-value is below 0.05).

S1.2.6 Measuring overlaps between prediction sets

To evaluate potential complementarity of two gene sets (e.g., A, B), we measure their overlap via the Jaccard index, $J(A, B) = \frac{A \cap B}{A \cup B}$. We compute the statistical significance of the overlap size using the hypergeometric test [22]. Because we run this test for multiple pairs of predictive models, we correct the p-values using the FDR correction. We choose 0.05 as the significance level (i.e., the overlap is statistically significantly large if the adjusted p-value is below 0.05).

S2 Selection of the best of the existing weighted dynamic features and the best of the proposed weighted dynamic features

Given the primary GenAge-based definition of aging- and non-aging-related gene labels, we compare the considered five existing weighted dynamic features (see *"The considered existing weighted dynamic features"* in the main paper) with respect to their ability to predict aging-related genes, in order to select the best existing weighted dynamic feature. For each of the five features, we first create a predictive model based on the logistics regression-based classifier (see *"Three considered classifiers"* in the main paper). Then, we evaluate the performance of the five predictive models based on average AUPR, average F1-score, average precision, and average recall. For details on how we compute these performance measures, see *"Evaluation measures"* in the main paper. We find that the best existing weighted dynamic feature is weighted degree centrality (Supplementary Fig. S1).

Similarly, given the primary GenAge-based definition of aging- and non-aging-related gene labels, we compare the considered 30 proposed weighted dynamic features (see *"The proposed weighted features"* in the main paper) with respect to their ability to predict aging-related genes, in order to select the best proposed weighted dynamic feature. For each of the 30 features, we first create a predictive model based on the logistics regression-based classifier (see *"Three considered classifiers"* in the main paper). Then, we evaluate the performance of the 30 predictive models based on average AUPR, average F1-score, average precision, and average recall. For details on how we compute these performance measures, see Supplementary Section S1.2.3 . We find that the best proposed weighted dynamic feature corresponds to the case when we use distributions of raw weights (i.e., approach 1 in Section *"The proposed weighted features"* of the main paper) of the second neighborhood type of a node, which we denote as "Diff-nobin-2" (Supplementary Fig. S1).

All methodology in this section is described when using the primary GenAge-based definition of aging- and non-aging-related gene labels. Everything is analogous when using the secondary GTEx-DAG definition.

S3 Overview of the NetWalk method

Given an age-specific gene expression data, the NetWalk algorithm works as follows. The NetWalk algorithm integrates the age-specific gene information with network topology immediately, by performing, from all nodes simultaneously, a random walk on the network biased by the age-specific gene information [23]. The output of the NetWalk algorithm is the entire static PPI network but with each PPI (i, j) in the network being assigned two bi-directional age-specific weights, one from node i to node j, and the other one from j to i. This is the final output of the NetWalk algorithm. To obtain a weighted age-specific subnetwork that each PPI has only one weight, we select the minimum of its two bi-directional weights as the final weight.

II Supplementary Tables

Supplementary Table S1: The number of predictive models that we test for each (sub)network for a given entire human PPI network data. In total, we consider $18 + 42 + 18 + 42 + 18 + 18 + 70 + 4 = 230$ models with respect to GenAge-based definition of aging- and non-aging-related gene labels for HPRD-based (sub)networks. Furthermore, because we apply PCA only on the best existing and the best proposed weighted dynamic features, we further consider $2 \times 1 \times 2 = 4$ models, which totals to 234 predictive models. The number of considered predictive models for HPRD-based (sub)networks when using GTEx-DAG-based definition of aging- and non-aging-related gene labels is also 234. Furthermore, we consider $234 - 2 \times 2 \times 3 - 2 \times 2 \times 3 = 210$ models for BioGRID-based (sub)networks. We remove 12 models for Induced-Dynamic and NetWalk-Dynamic because we do not run DGDV and GoT on them. Thus, in total, we consider $234 + 234 + 210 = 678$ predictive models in this study.

Network type	Network name	Feature count	Dimensionality reduction choices	Classifier count	Predictive model count
Unweighted static	Entire	3	2	3	18
Unweighted dynamic	Induced-Dynamic	7	2	3	42
Unweighted static	Induced-Static	3	2	3	18
Unweighted dynamic	NetWalk-Dynamic	7	2	3	42
Unweighted static	NetWalk-Static	3	2	3	18
Unweighted static	NetWalk-Static*	3	2	3	18
Weighted dynamic	wNetWalk-Dynamic	35	1	2	70
Weighted static	wNetWalk-Static*	1	2	2	4

Supplementary Table S2: The selected best predictive model for each (sub)network with respect to the GTEx-DAG-based aging- and non-aging-related gene labels, and when using HPRD. The model is represented as "X+Y+Z", where X represents the selected feature, Y represents the selected dimensionality reduction choice (i.e., None or PCA), and Z represents the selected classifier. For dimensionality reduction choices, "None" means the selected feature is in its full version, and "PCA" means the selected feature is in its PCA-reduced version.

Entire	Induced-Dynamic	Induced-Static
30BPIs + PCA + SVM-rbf	GDC + None + NB	SGDV + PCA + NB
NetWalk-Dynamic	NetWalk-Static	NetWalk-Static*
DegC + None + LR	SGDV + PCA + LR	SGDV + PCA + LR
wNetWalk-Dynamic	wNetWalk-Static*	
Diff-bin-1 + PCA + LR	Static-bin-1 + None + LR	

Supplementary Table S3: The selected best predictive model for each (sub)network with respect to the GenAge-based aging- and non-aging-related gene labels, and when using BioGRID. The model is represented as "X+Y+Z", where X represents the selected feature, Y represents the selected dimensionality reduction choice (i.e., None or PCA), and Z represents the selected classifier. For dimensionality reduction choices, "None" means the selected feature is in its full version, and "PCA" means the selected feature is in its PCA-reduced version.

Entire	Induced-Dynamic	Induced-Static
UniNet + PCA + LR	GDC + None + NB	30BPIs + None + LR
NetWalk-Dynamic	NetWalk-Static	NetWalk-Static*
CentraMV + None + NB	UniNet + PCA + LR	30BPIs + None + LR
wNetWalk-Dynamic	wNetWalk-Static*	
Diff-nobin-cor-2 + None + LR	Static-nobin-2 + None + LR	

Supplementary Table S4: The sizes of the eight considered BioGRID-based (sub)networks. The size of a dynamic subnetwork is the average over its 37 (or 36, depending on whether the considered dynamic subnetwork has 37 or 36 snapshots) subnetwork snapshots. The two numbers delimited by ";" are node and edge counts of the corresponding (sub)network.

Entire	Induced-Dynamic	Induced-Static	NetWalk-Dynamic
18,928; 484,146	8,452; 192,578	11,342; 278,319	11,623; 243,656
NetWalk-Static	NetWalk-Static*	wNetWalk-Dynamic	wNetWalk-Static*
14,011; 303,384	15,141; 303,384	18,928; 484,146	18,928; 484,146

III Supplementary Figures

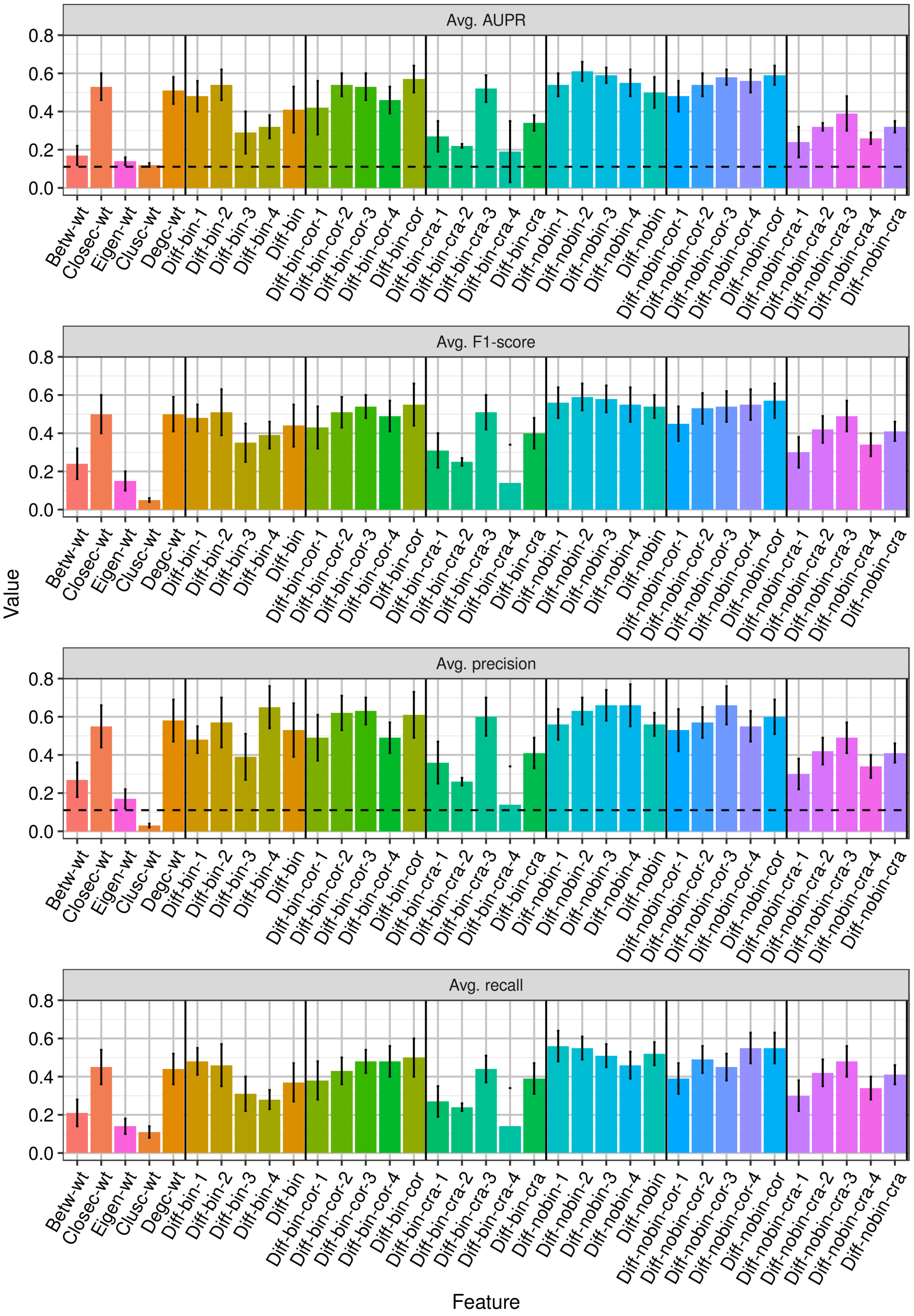

Supplementary Fig. S1: Prediction accuracy of the logistic regression-based prediction models corresponding to the considered existing weighted features (leftmost five bars in a panel) and proposed dynamic weighted features (rightmost 30 bars in a panel) for the HPRD-based *w*NetWalk-Dynamic, in terms of average AUPR, average F-score, average precision, and average recall, when using the primary GenAge-based definition of aging- and non-aging-related gene labels.

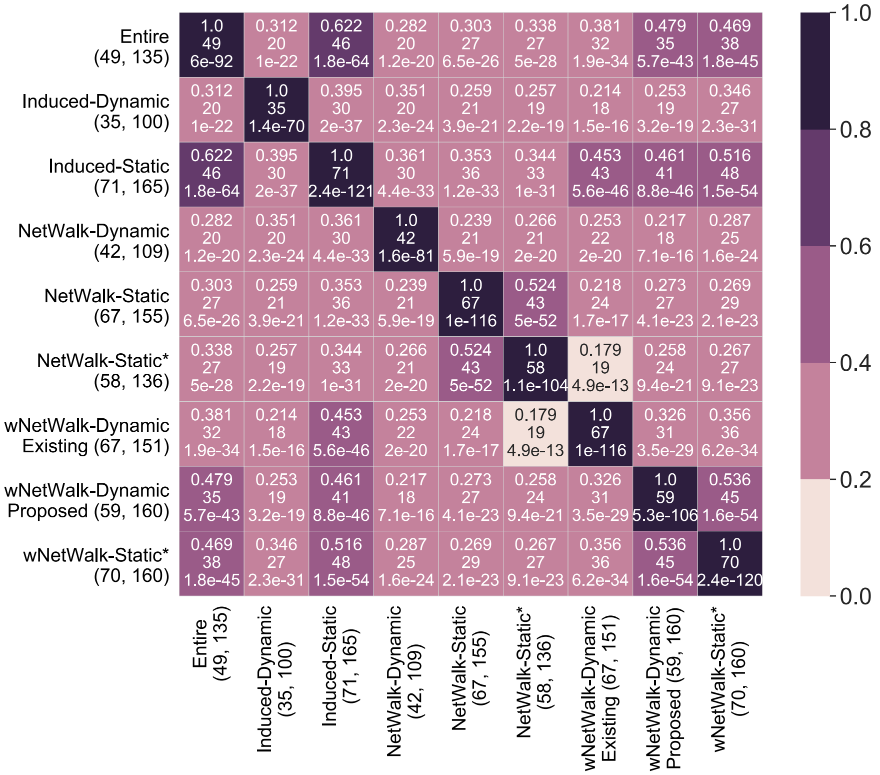

Supplementary Fig. S2: Overlaps (expressed as Jaccard indices) between novel predictions of the eight considered (sub)networks, each under its best predictive model, when using the primary GenAge-based definition of aging- and non-aging-related gene labels, and when using HPRD. On the x- and y-axes, the numbers in the parentheses after the name of each subnetwork are the number of true positive predictions (i.e., known aging-related genes) and the number of total predictions. Within each table/matrix cell, the number on the first line is the Jaccard index of the overlap between the corresponding pair of subnetworks; the number on the second line is the raw overlap size, i.e., the actual count of true positives that are in the overlap between the two subnetworks; the number on the third line is the adjusted p-value of the overlap with respect to the hypergeometric test. The darker the color of a given cell, the larger the Jaccard index value, i.e., the higher the overlap. Note that we have two groups of results for wNetWalk-Dynamic, i.e., wNetWalk-Dynamic Existing and wNetWalk-Dynamic Proposed. The former corresponds to using the best existing weighted dynamic feature on wNetWalk-Dynamic, and the latter corresponds to using the best proposed weighted dynamic feature on wNetWalk-Dynamic. For results when using BioGRID instead of HPRD, see Supplementary Fig. S6.

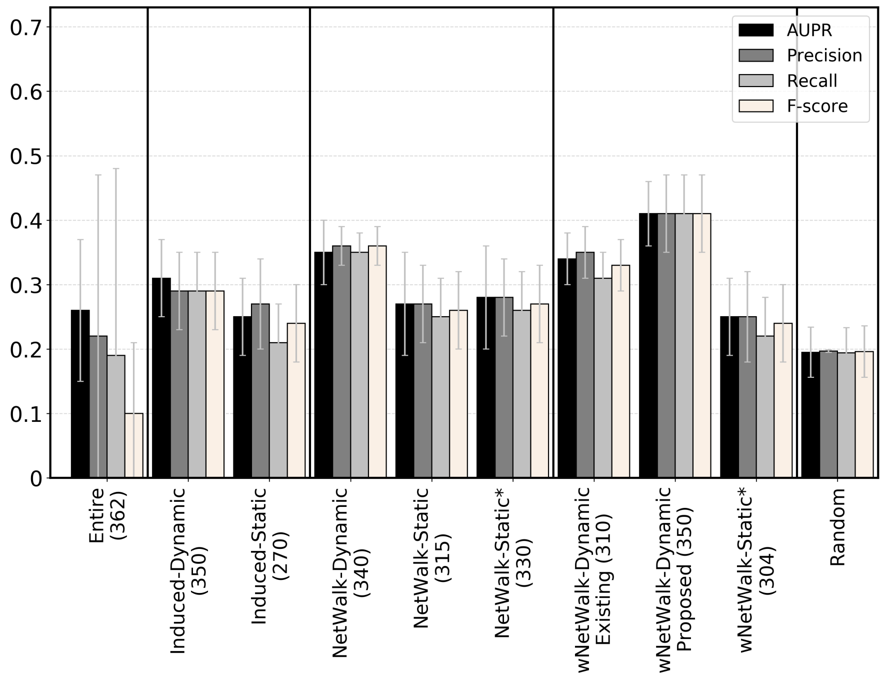

Supplementary Fig. S3: Prediction accuracy in terms of AUPR, precision, recall, and F-score for the eight (sub)networks, each under its best predictive model, when using the secondary GTEx-DAG-based definition of aging- and non-aging-related gene labels, and when using HPRD. On the x-axis, the number in the parenthesis after the name of each (sub)network corresponds to the number of predictions. Note that we have two groups of results for wNetWalk-Dynamic, i.e., wNetWalk-Dynamic Existing and wNetWalk-Dynamic Proposed, which we already explained in the caption of Supplementary Fig. S2.

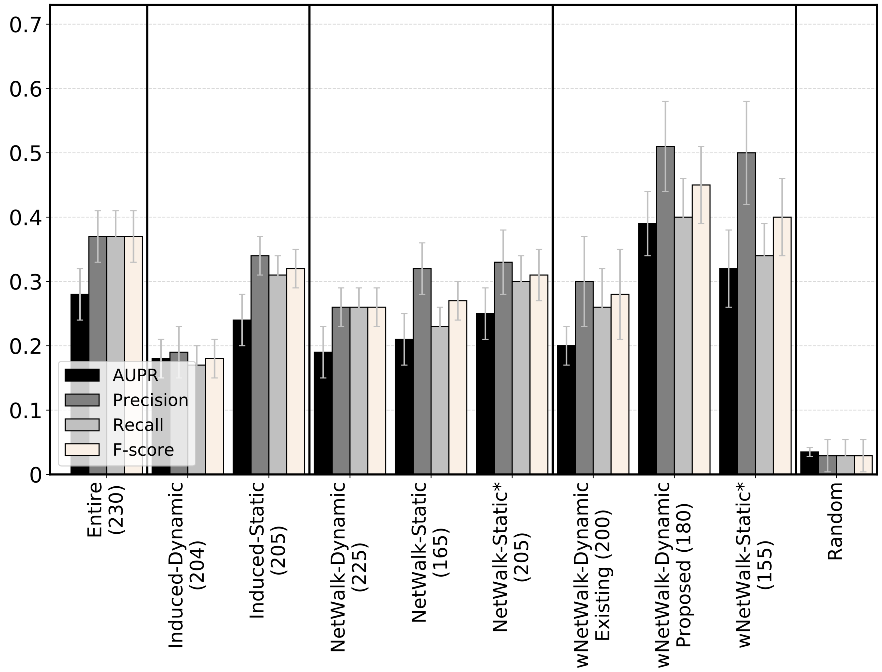

Supplementary Fig. S4: Prediction accuracy in terms of AUPR, precision, recall, and F-score for the eight (sub)networks, each under its best predictive model, when using the primary GenAge-based definition of aging- and non-aging-related gene labels, and when using BioGRID. On the x-axis, the number in the parenthesis after the name of each (sub)network corresponds to the number of predictions. Note that we have two groups of results for wNetWalk-Dynamic, i.e., wNetWalk-Dynamic Existing and wNetWalk-Dynamic Proposed. The former corresponds to using the best existing weighted dynamic feature on wNetWalk-Dynamic, and the latter corresponds to using the best proposed weighted dynamic feature on wNetWalk-Dynamic. Note that we have two groups of results for wNetWalk-Dynamic, i.e., wNetWalk-Dynamic Existing and wNetWalk-Dynamic Proposed. The former corresponds to using the best existing weighted dynamic feature on wNetWalk-Dynamic, and the latter corresponds to using the best proposed weighted dynamic feature on wNetWalk-Dynamic.

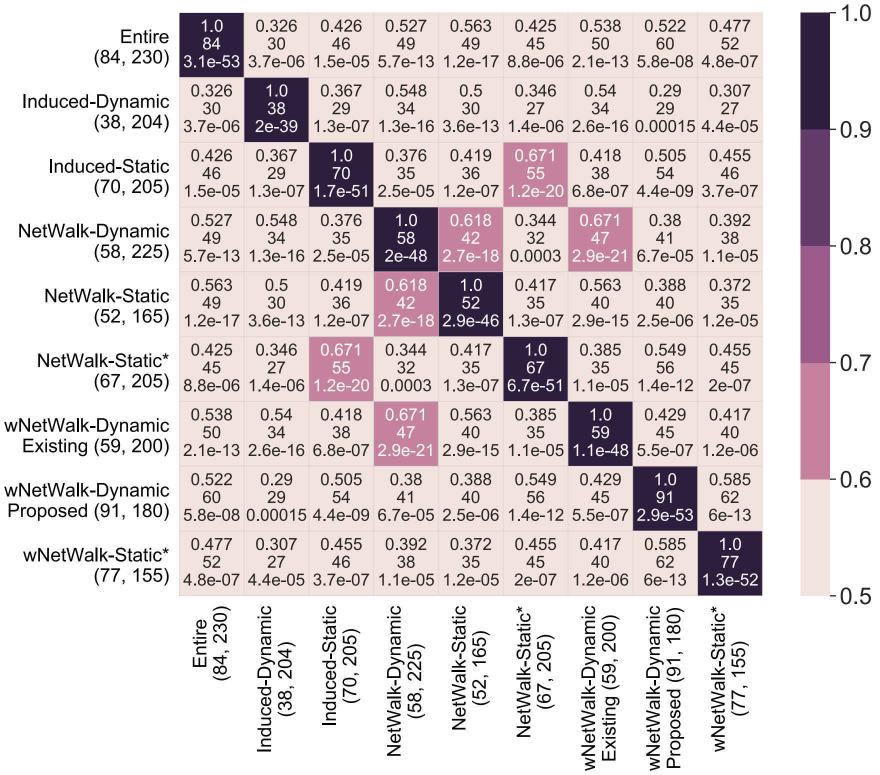

Supplementary Fig. S5: Overlaps (expressed as Jaccard indices) between true positive predictions of the eight considered (sub)networks, each under its best predictive model, when using the primary GenAge-based definition of aging- and non-aging-related gene labels, and when using BioGRID. On the x- and y-axes, the numbers in the parentheses after the name of each subnetwork are the number of true positive predictions (i.e., known aging-related genes) and the number of total predictions. Within each table/matrix cell, the number on the first line is the Jaccard index of the overlap between the corresponding pair of subnetworks; the number on the second line is the raw overlap size, i.e., the actual count of true positives that are in the overlap between the two subnetworks; the number on the third line is the adjusted p-value of the overlap with respect to the hypergeometric test. The darker the color of a given cell, the larger the Jaccard index value, i.e., the higher the overlap. Note that we have two groups of results for wNetWalk-Dynamic, i.e., wNetWalk-Dynamic Existing and wNetWalk-Dynamic Proposed, which we already explained in the caption of Supplementary Fig. S4.

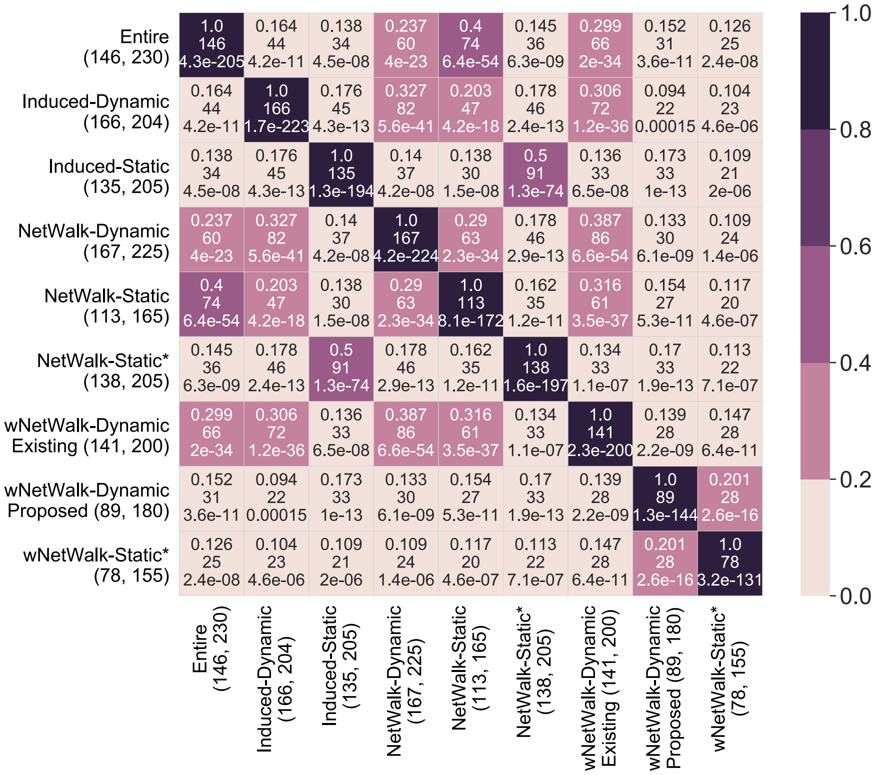

Supplementary Fig. S6: Overlaps (expressed as Jaccard indices) between novel predictions of the eight considered (sub)networks, each under its best predictive model, when using the primary GenAge-based definition of aging- and non-aging-related gene labels, and when using BioGRID. On the x- and y-axes, the numbers in the parentheses after the name of each subnetwork are the number of true positive predictions (i.e., known aging-related genes) and the number of total predictions. Within each table/matrix cell, the number on the first line is the Jaccard index of the overlap between the corresponding pair of subnetworks; the number on the second line is the raw overlap size, i.e., the actual count of true positives that are in the overlap between the two subnetworks; the number on the third line is the adjusted p-value of the overlap with respect to the hypergeometric test. The darker the color of a given cell, the larger the Jaccard index value, i.e., the higher the overlap. Note that we have two groups of results for wNetWalk-Dynamic, i.e., wNetWalk-Dynamic Existing and wNetWalk-Dynamic Proposed, which we already explained in the caption of Supplementary Fig. S4.

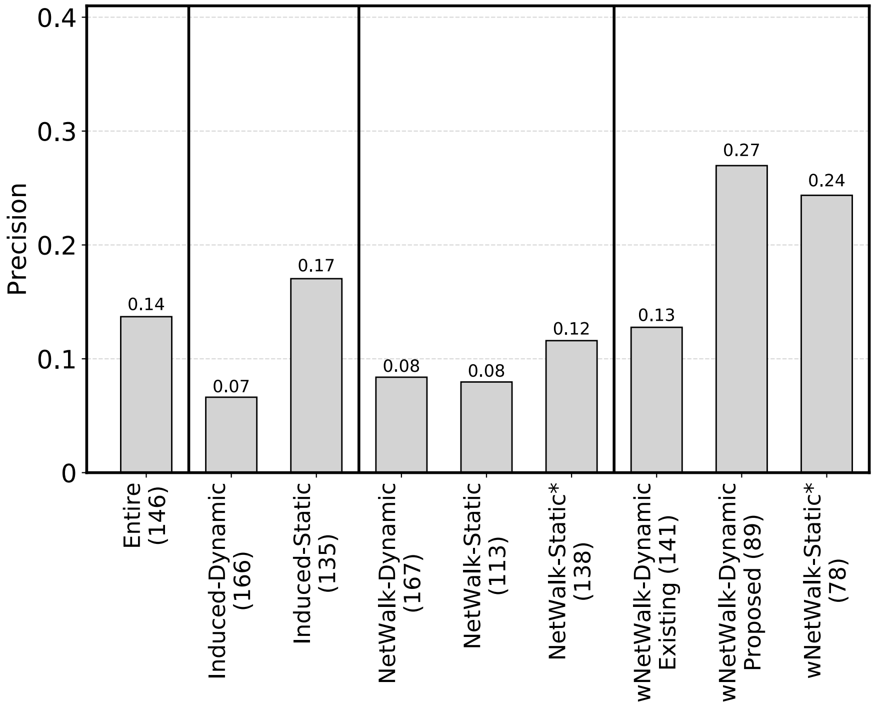

Supplementary Fig. S7: Cancer-related validation of the novel aging-related gene predictions for the eight considered (sub)networks, each under its best predictive model, when using the primary GenAge-based definition of aging- and non-aging-related gene labels, and when using BioGRID. On the x-axis, the number in the parentheses after the name of each (sub)network is the number of the novel predictions. The precision score of a (sub)network is the percentage of novel predictions that are validated by the cancer-related data. Note that we have two groups of results for wNetWalk-Dynamic, i.e., wNetWalk-Dynamic Existing and wNetWalk-Dynamic Proposed, which we already explained in the caption of Supplementary Fig. S4.

References

[1] Hulovatyy, Y., Chen, H., Milenković, T.: Exploring the structure and function of temporal networks with dynamic graphlets. Bioinformatics **31**(12), 171–180 (2015)

[2] Aparício, D., Ribeiro, P., Silva, F.: Graphlet-orbit transitions (GoT): A fingerprint for temporal network comparison. PLOS ONE **13**(10), 0205497 (2018)

[3] Milenković, T., Memišević, V., Bonato, A., Pržulj, N.: Dominating biological networks. PLOS ONE **6**(8), 23016 (2011)

[4] Faisal, F.E., Milenković, T.: Dynamic networks reveal key players in aging. Bioinformatics **30**(12), 1721–1729 (2014)

[5] Li, Q., Milenković, T.: Supervised prediction of aging-related genes from a context-specific protein interaction subnetwork. IEEE International Conference on Bioinformatics and Biomedicine (BIBM) **pp**, 130–137 (2019)

[6] Li, Q., Milenković, T.: Supervised prediction of aging-related genes from a context-specific protein interaction subnetwork†. arXiv preprint arXiv:1908.08135 (2021). Note: This paper is under review, and it is an extended version of the IEEE BIBM 2019 paper with the same name.

[7] Milenković, T., Pržulj, N.: Uncovering biological network function via graphlet degree signatures. Cancer Informatics **6**, 680 (2008)

[8] Kerepesi, C., Daróczy, B., Sturm, Á., Vellai, T., Benczúr, A.: Prediction and characterization of human ageing-related proteins by using machine learning. Scientific Reports **8**(1), 4094 (2018)

[9] Freitas, A.A., Vasieva, O., de Magalhães, J.P.: A data mining approach for classifying DNA repair genes into ageing-related or non-ageing-related. BMC Genomics **12**(1), 27 (2011)

[10] Opsahl, T., Agneessens, F., Skvoretz, J.: Node centrality in weighted networks: Generalizing degree and shortest paths. Social Networks **32**(3), 245–251 (2010)

[11] Barrat, A., Barthélemy, M., Pastor-Satorras, R., Vespignani, A.: The architecture of complex weighted networks. Proceedings of the National Academy of Sciences **101**(11), 3747–3752 (2004)

[12] Saramäki, J., Kivelä, M., Onnela, J.-P., Kaski, K., Kertész, J.: Generalizations of the clustering coefficient to weighted complex networks. Phys. Rev. E **75**, 027105 (2007)

[13] Newman, M.E.J.: Analysis of weighted networks. Phys. Rev. E **70**, 056131 (2004)

[14] Subramanian, J., Simon, R.: Overfitting in prediction models–is it a problem only in high dimensions? Contemporary Clinical Trials **36**(2), 636–641 (2013)

[15] Maaten, L.v.d., Hinton, G.: Visualizing data using t-SNE. Journal of Machine Learning Research **9**(Nov), 2579–2605 (2008)

[16] Yu, H.-F., Huang, F.-L., Lin, C.-J.: Dual coordinate descent methods for logistic regression and maximum entropy models. Machine Learning **85**(1-2), 41–75 (2011)

[17] Fan, R.-E., Chang, K.-W., Hsieh, C.-J., Wang, X.-R., Lin, C.-J.: LIBLINEAR: A library for large linear classification. Journal of Machine Learning Research **9**(Aug), 1871–1874 (2008)

[18] Chan, T.F., Golub, G.H., LeVeque, R.J.: Updating formulae and a pairwise algorithm for computing sample variances. In: COMPSTAT 1982 5th Symposium Held at Toulouse 1982, pp. 30–41 (1982). Springer

[19] Pedregosa, F., Varoquaux, G., Gramfort, A., Michel, V., Thirion, B., Grisel, O., Blondel, M., Prettenhofer, P., Weiss, R., Dubourg, V., Vanderplas, J., Passos, A., Cournapeau, D., Brucher, M., Perrot, M., Duchesnay, E.: Scikit-learn: Machine learning in Python. Journal of Machine Learning Research **12**, 2825–2830 (2011)

[20] Wilcoxon, F.: Individual comparisons by ranking methods. Breakthroughs in Statistics, 196–202 (1992)

[21] Benjamini, Y., Hochberg, Y.: Controlling the false discovery rate: a practical and powerful approach to multiple testing. Journal of the Royal Statistical Society: series B (Methodological) **57**(1), 289–300 (1995)

[22] Rivals, I., Personnaz, L., Taing, L., Potier, M.-C.: Enrichment or depletion of a GO category within a class of genes: which test? Bioinformatics **23**(4), 401–407 (2007)

[23] Komurov, K., White, M.A., Ram, P.T.: Use of data-biased random walks on graphs for the retrieval of context-specific networks from genomic data. PLOS Computational Biology **6**(8), 1–10 (2010)
